# Changes in China’s lakes: climate and human impacts

**DOI:** 10.1093/nsr/nwz103

**Published:** 2019-07-25

**Authors:** Shengli Tao, Jingyun Fang, Suhui Ma, Qiong Cai, Xinyu Xiong, Di Tian, Xia Zhao, Leqi Fang, Heng Zhang, Jiangling Zhu, Shuqing Zhao

**Affiliations:** 1 Institute of Ecology, College of Urban and Environmental Sciences, and Key Laboratory for Earth Surface Processes of the Ministry of Education, Peking University, Beijing 100871, China; 2 State Key Laboratory of Vegetation and Environmental Change, Institute of Botany, Chinese Academy of Sciences, Beijing 100093, China; 3 School of Electronics Engineering and Computer Science, Peking University, Beijing 100871, China

**Keywords:** inland water, sustainability, population, Landsat

## Abstract

Lakes have played a critical role in providing water and ecosystem services for people and other organisms in China for millennia. However, accelerating climate change and economic boom have resulted in unprecedented changes in these valuable lakes. Using Landsat images covering the entity of the country, we explored the changes in China’s lakes and the associated driving forces over the last 30 years (i.e. mid-1980s to 2015). We discovered that China’s lakes have changed with divergent regional trends: in the sparsely populated Tibetan Plateau, lakes are abundant and the lake area has increased dramatically from 38 596 to 46 831 km^2^ (i.e. increased by 8235 km^2^, or 21.3%), whereas, in the densely populated northern and eastern regions, lakes are relatively scarce and the lake area has decreased from 36 659 to 33 657 km^2^ (i.e. decreased by 3002 km^2^, or 8.2%). In particular, severe lake decreases occurred in the Mongolia-Xinjiang Plateau and the Eastern Plain (−2151 km^2^). Statistical analyses indicated that climate was the most important factor controlling lake changes in the Tibetan Plateau, the Yun-Gui Plateau and the Northeast Plain. However, the strength of climatic control on lake changes was low in the Eastern Plain and the Mongolia-Xinjiang Plateau, where human activities, e.g. impoldering, irrigation and mining, have caused serious impacts on lakes. Further lake changes will exacerbate regional imbalances between lake resources and population distribution, and thus may increase the risk of water-resource crises in China.

## INTRODUCTION

**Figure 1.**
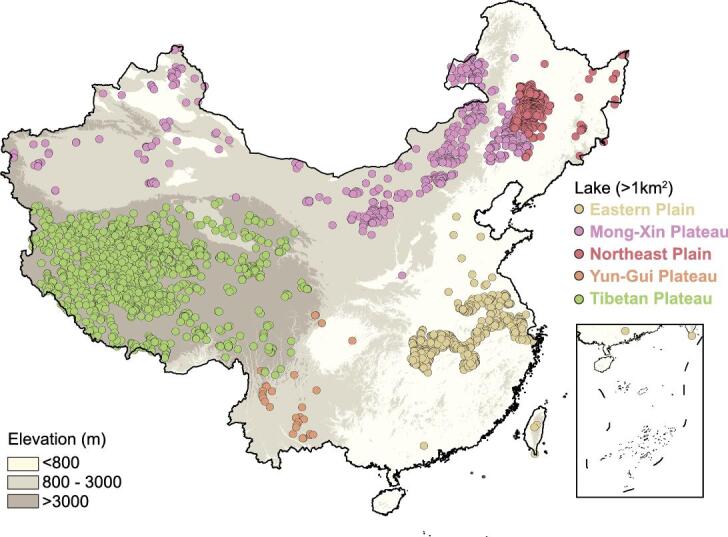
Distribution of lakes with areas greater than 1 km^2^ in China.

China is a water-deficit country, where 18.5% of the world’s population shares only 7.7% of the global freshwater [1–3]. Lakes in China represent one of the most treasured water resources, providing a wide range of services for Chinese ecological and social systems [4–9]. Owing to the tremendous differences in geography and climate, China’s lakes are very diverse and have traditionally been categorized into five lake regions [4,5]—the Eastern Plain, the Mong-Xin (Mongolia-Xinjiang) Plateau, the Northeast Plain, the Yun-Gui (Yunnan-Guizhou) Plateau and the Tibetan Plateau lake regions (Fig. 1; for details, see Methods). These five lake regions have functioned differently for millennia, nourishing the people and other organisms in China. For instance, the Mong-Xin Plateau lake region is an essential part of the Mongolian Plateau, where lakes are important to the grasslands, local people and endangered species [6]; the Eastern Plain lake region contains all five of the largest freshwater lakes in China, which, together with the Yangtze River, form one of the most biologically diverse wetlands in the world [7]. In the Tibetan Plateau, many lakes are deemed sacred by local people, who proclaim the vast blue waters the holy lands of their religion [8] (for detailed descriptions on these five lake regions, please see Supplementary Text 1).

However, in the context of the global inland water changes [10–13], China’s lakes have also been exposed to severe threats and are exceptional for the acuteness of these issues (see Supplementary Text 2 for a short review on historic changes in China’s lakes). Since the 1980s, changes in climate have been pronounced in China [14]. The rapid economic and population growth, which occurred at nearly the same time, acted against the backdrop of climatic changes, and eventually caused dramatic lake changes.

Securing a sustainable future for China’s lakes is an urgent task facing both scientists and policy makers, and this effort requires a diagnosis of the lake changes and their causes. Although studies focusing on water changes have been conducted for different periods and regions [6,10,15–23], controversies still exist over the extent of the lake changes and the associated driving forces. Using Landsat images covering the entirety of China (see Methods), in combination with our past 20 years of efforts devoted to lake research [6,15–19], we presented a nationwide assessment on China’s lake changes and their driving forces over the past 30 years (from the mid-1980s to 2015). Existing studies on the recent and historic changes in China’s lakes were also reviewed (Supplementary Texts 3 and 4). We further developed a database on China’s lakes (Supplementary Database), hoping to facilitate future researches on this topic.

## RESULTS AND DISCUSSION

### Changes in lakes >1 km^2^ between the mid-1980s and 2015

**Figure 2.**
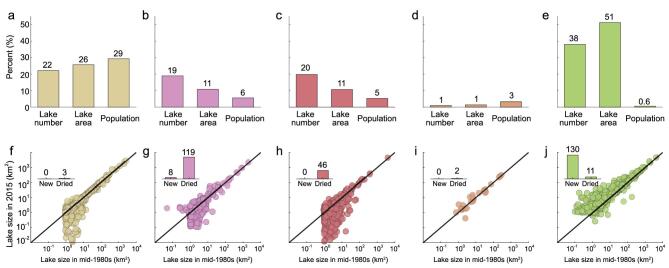
Lake distribution and lake changes between the mid-1980s and 2015 for all five lake regions in China. (a) and (f), Eastern Plain; (b) and (g), Mong-Xin Plateau; (c) and (h), Northeast Plain; (d) and (i), Yun-Gui Plateau; (e) and (j), Tibetan Plateau. (a) –(e), percentages of lake number, lake area and population relative to the national totals for each lake region. (f) –(j), lake changes between the mid-1980s and 2015 (shown on a logarithmic scale). The inset bar graphs in (f)–(j) show the number of newly formed and dried-up lakes.

We first quantified the changes in the number and area of all lakes >1 km^2^. According to previous studies [4,5], these lakes were categorized into three classes: small (1–10 km^2^), medium (10–50 km^2^) and large lakes (>50 km^2^). We identified 2799 lakes with a water area >1 km^2^ in the mid-1980s (Fig. 1), covering a total surface area of 75 255 km^2^ (Table 1). The lake distribution among the five lake regions was highly uneven and was poorly matched with human demand (Fig. 2a–e and Table 1): the Tibetan lake region contained 51% of the lake resources but less than 1% of the total population. The remaining four lake regions, however, contained 44% of the population but only 49% of the lake resources (see Methods for the calculation of the population for each lake region).

**Figure 3.**
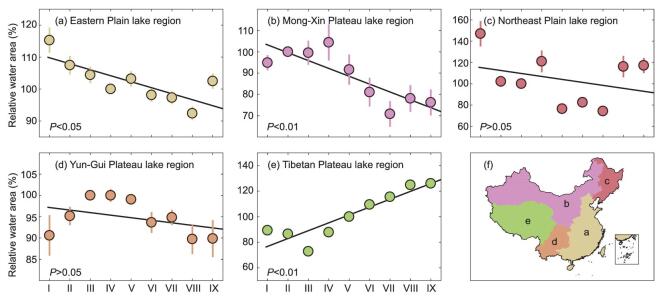
Temporal changes in China's lakes. (a)–(e), temporal overall changes in medium and large lakes (>10 km^2^) from the mid-1980s to 2015 for the five lake regions. The locations of the five lake regions are shown in (f). The overall lake area for each lake region is presented using the relative water area (RWA) method (see Methods). Periods I–IX on the *x*-axis indicate the periods of 1985–1990 (I), 1991–1993 (II), 1994–1997 (III), 1998–2000 (IV), 2001–2003 (V), 2004–2007 (VI), 2008–2010 (VII), 2011–2013 (VIII) and 2014–2015 (IX).

**Table 1.** Changes in the number and area of all lakes >1 km^2^ in China between the mid-1980s and 2015.

		Lake number	Lake area (km^2^)	Lake number	Lake area	Number of	Number of	Change in	Change in lake	Change in	Change in
Region	Lake class	in mid-1980s	in mid-1980s	in 2015	(km^2^) in 2015	dried-up lakes	new lakes	lake number	number (%)	lake area	lake area (%)
**National**	1–10	2115	5674.1	2072	5369.7	168	125	−43	−2.0	−304.4	−5.4
	10–50	411	7165.6	417	8493.4	7	13	6	1.5	1327.8	18.5
	>50	273	62 415.5	267	66 624.2	6	0	−6	−2.2	4208.7	6.7
	All	2799	75 255.2	2756	80 487.3	181	138	−43	−1.5	5232.1	7.0
**Eastern Plain**	1–10	483	1572.8	481	1330.8	2	0	−2	−0.4	−242.0	−15.4
	10–50	89	1916.5	88	1755.6	1	0	−1	−1.1	−160.9	−8.4
	>50	49	15 894.5	49	15 076.0	0	0	0	0	−818.5	−5.1
	All	621	19 383.9	618	18 162.4	3	0	−3	−0.5	−1221.5	−6.3
**Mong-Xin Plateau**	1–10	478	1260.4	373	824.6	110	5	−105	−22.0	−435.7	−34.6
	10–50	30	463.2	28	485.9	5	3	−2	−6.7	22.7	4.9
	>50	23	6449.6	19	5933.0	4	0	−4	−17.4	−516.6	−8.0
	All	531	8173.1	420	7243.6	119	8	−111	−20.9	−929.6	−11.4
**Northeast Plain**	1–10	485	1251.5	440	714.3	45	0	−45	−9.3	−537.2	−42.9
	10–50	56	948.9	55	697.9	1	0	−1	−1.8	−251.0	−26.5
	>50	14	5844.8	14	5786.4	0	0	0	0	−58.4	−1.0
	All	555	8045.2	509	7198.6	46	0	−46	−8.3	−846.6	−10.5
**Yun-Gui Plateau**	1–10	15	46.1	13	44.0	2	0	−2	−13.3	−2.1	−4.6
	10–50	8	192.3	8	179.1	0	0	0	0	−13.2	−6.9
	>50	4	818.5	4	829.1	0	0	0	0	10.6	1.3
	All	27	1056.9	25	1052.2	2	0	−2	−7.4	−4.7	−0.4
**Tibetan Plateau**	1–10	654	1543.3	765	2456.0	9	120	111	17.0	912.6	59.1
	10–50	228	3644.6	238	5374.8	0	10	10	4.4	1730.2	47.5
	>50	183	33 408.1	181	38 999.7	2	0	−2	−1.1	5591.7	16.7
	All	1065	38 596.0	1184	46 830.5	11	130	119	11.2	8234.5	21.3

Unfortunately, this huge regional disparity between the distributions of lake resources and population has become further exacerbated over the past 30 years. The lake area has increased only in the Tibetan Plateau and has decreased pervasively in the other lake regions (Table 1, Fig. 2f–j and Supplementary Fig. 1). Specifically, the lake area in the Eastern Plain region has decreased dramatically by 1222 km^2^ from 19 384 to 18 162 km^2^. The Mong-Xin Plateau has suffered the most notable decrease in lake numbers, with a net loss of 111 lakes (from 531 to 420). The decreases in the lake area in the Northeast Plain (−847 km^2^) and the Yun-Gui Plateau (−5 km^2^) were relatively weaker but were still considerable. In sharp contrast to these four lake regions, the number of lakes in the Tibetan Plateau has increased from 1065 to 1184 (+119) and the lake area has increased from 38 596 to 46 831 km^2^ (+8235 km^2^) (Table 1). These tremendous lake increases in the Tibetan Plateau have substantially offset the decreases in the other four lake regions, resulting in a small loss in the country’s total number of lakes (from 2799 to 2756) and, unexpectedly, a net national increase of 5232 km^2^ in lake area (from 75 255 to 80 487; Table 1).

### Temporal changes in lakes >10 km^2^ between the mid-1980s and 2015

To illustrate the temporal dynamics of China’s lakes, we then examined the 30-year changes in all 684 medium (10–50 km^2^) and large lakes (>50 km^2^), as they comprise most of the total lake area. Among the 421 medium and large lakes in the Tibetan Plateau, 382 lakes (91%) increased and 39 (9%) decreased. Among these lakes, the changes in 300 of the growing lakes and 9 of the shrinking lakes were statistically significant (Supplementary Fig. 2). In contrast, 106 (77%) of the 138 medium and large lakes in the Eastern Plain exhibited decreasing trends (23 with significant changes) and only 32 (23%) increased (2 with significant changes) (Supplementary Fig. 3). For the Mong-Xin Plateau, 55% of the medium and large lakes decreased and 45% increased (mostly glacier-fed lakes in Xinjiang) (Supplementary Fig. 4). For the Northeast Plain, the number of lakes with a significant increase in area (seven lakes, 10%) was slightly less than that with a significant decline (nine lakes, 13%) (Supplementary Fig. 5). There were only 12 medium and large lakes in the Yun-Gui Plateau, 4 of which showed a significant decrease in water area (Supplementary Fig. 6).

The temporal changes of the 684 medium and large lakes were further investigated by each lake region, using the relative water area method (RWA), which presents the overall changes in all the lakes in a region [6,19] (see Methods). Figure 3 shows the temporal changes in the RWAs for the five lake regions. The RWAs increased by 37% (*P* < 0.01; Fig. 3e) in the Tibetan Plateau but decreased notably in the Eastern Plain (Fig. 3a; *P* < 0.05) and the Mong-Xin lake regions (Fig. 3b; *P* < 0.01), although the decreasing trends have partially reversed recently. Both in the Northeast Plain (Fig. 3c) and in the Yun-Gui Plateau (Fig. 3d), the RWAs tended to decrease but fluctuated greatly. In summary, the observed temporal changes aggravated the regional disparity in lake distribution, especially the large difference between the Tibetan Plateau and the other four lake regions.

### Driving forces of lake changes

Climate change is widely believed to be the dominant driver of historic changes in China’s lakes (see Supplementary Text 2 for a brief review). An important question, then, is whether climate factors are controlling the lake changes in the recent decades.

**Figure 4.**
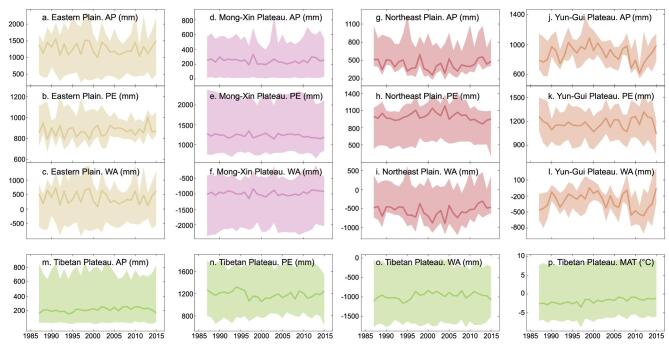
Changes in climatic conditions in the five lake regions between the mid-1980s and 2015. (a)–(c), Eastern Plain; (d)–(f), Mong-Xin Plateau; (g)–(i), Northeast Plain; (j)–(l), Yun-Gui Plateau; (m)–(p), Tibetan Plateau. Annual precipitation (AP), annual pan evaporation (PE) and annual water availability (WA, calculated as AP – PE) are shown for all five lake regions. Mean annual temperature (MAT) is shown additionally for the Tibetan Plateau lake region. The shaded area in each subfigure shows the range of climatic conditions over all medium and large lakes, and the bold line shows the averaged condition.

From the mid-1980s to 2015, annual precipitation (AP) and annual pan evaporation (PE) over Eastern Plain lakes have been fluctuating. As a result, water availability (WA, calculated as the difference between AP and PE) showed no obvious changes (Fig. 4a–c), which seems unable to explain the observed lake decrease (Fig. 3a). As for the lakes in the Mong-Xin Plateau, rainfall input (ranging from 7 to 842 mm/yr, with an average of 245 mm/yr) is much less than the evaporation loss (ranging from 653 to 2381 mm/yr and averaging 1225 mm/yr), but WA has been relatively stable (Fig. 4d–f), in contrast to the startling lake decrease that occurred in the plateau (Fig. 3b). WA has changed significantly in the Northeast Plain and the Yun-Gui Plateau, especially since 2000 (Fig. 4g–l), tracked closely by their RWAs (Fig. 3c and d). The Tibetan Plateau has received a higher amount of rainfall and reduced PE since around 1995 (Fig. 4m and n), which were reported as important reasons for the lake increase [21,24]. Meanwhile, rapid warming is occurring in the plateau (Fig. 4p), resulting in additional water for lakes from melting glaciers, snow and permafrost [25–27].

**Figure 5.**
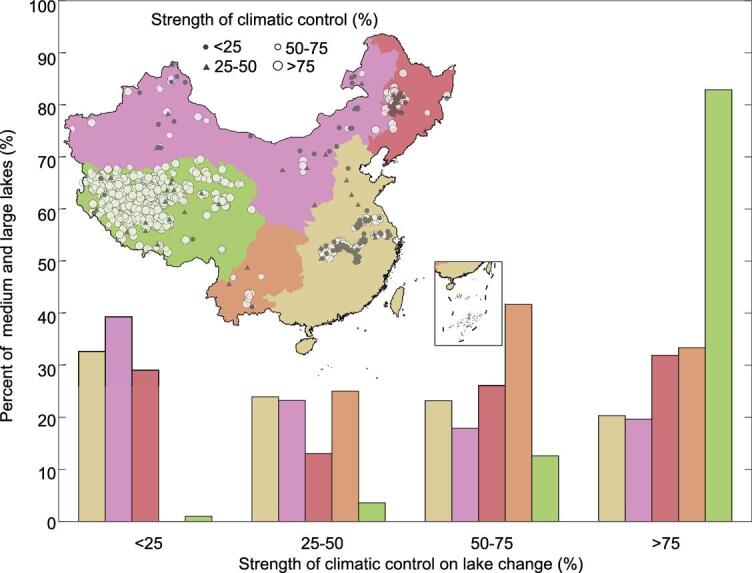
Strengths of climatic control on lake changes for all medium and large lakes (>10 km^2^) in China. The five lake regions were shown in different colors. The strength of climatic control was calculated by decomposing the variance of the linear fitting model between climate factors and lake area. Water availability (WA) was used as a climate factor for non-Tibetan lakes, and WA and mean annual temperature (MAT) were used for Tibetan lakes. The inset figure shows the spatial pattern of the strength of climatic control on each medium and large lake. The bar figure shows the regional statistics.

To quantify the influences of climate change on lakes, we performed general linear model fitting between climate factors and lake area for each medium and large lake. The proportion of variance in the lake area explained by climate was further quantified by decomposing the variance of the linear model. We chose WA as the climate factor for all five lake regions, since it represents the combined effect of AP and PE. For the Tibetan lakes, an additional factor—mean annual temperature—was used, because temperature is a good surrogate for meltwater from glaciers, snow and permafrost (see Methods). The results showed large regional differences in the strength of climatic control on lake changes (Fig. 5). Climate explained more than half of the lake variances for 95% medium and large lakes (402 out of 421) in the Tibetan Plateau, followed by 75% (9 out of 12) in the Yun-Gui Plateau and 57% (40 out of 70) in the Northeast Plain. However, the strength of climatic control was low in the Eastern Plain and the Mong-Xin Plateau. Only 46% (60 out of 138) medium and large lakes were well explained by climate in the Eastern Plain, and the number decreased to 38% (21 out of 56) in the Mong-Xin Plateau. These results implied that humans rather than climate might be the primary cause of the severe lake decreases that occurred in the Eastern Plain and the Mong-Xin Plateau.

Although our analyses did not consider groundwater and river flow, the above results generally coincided with previous research, in which climate impacts on lakes were emphasized in the Tibetan Plateau, the Yun-Gui Plateau and the Northeast Plain, but human influences attracted much more attention in the Mong-Xin Plateau and the Eastern Plain (see Supplementary Text 3 for a brief review). Using high-resolution land-use maps, and by reviewing existing research (Supplementary Text 3), we further explored the effects of human activities on Mong-Xin and Eastern Plain lakes.

The Mong-Xin Plateau is an important part of the Eurasian Steppe. However, large areas of the grassland have been reclaimed for agricultural fields in the past decades [28,29]. We used annual land-use maps at 300-m resolution between 1992 and 2015 to check the severity of the reclamation and found an increase in the cropland area as large as 3.74×10^4^ km^2^ (Supplementary Fig. 7). Croplands need to be irrigated but the plateau receives limited rainfall (Fig. 4d–f). Consequently, rivers have been dammed or diverted to provide irrigation water, causing drastic lake shrinkages in the lower reaches of the rivers [6,19,30; Supplementary Text 3]. Moreover, the plateau happens to be one of the world’s largest coal-mining regions [6,28,29]. Mining destroyed groundwater aquifers, intercepted rivers and caused the rapid drying-up of lakes around the mining sites [6,31]. By analysing existing case studies, we found direct evidence for the effects of irrigation and mining on many lakes in the plateau, such as Huangqihai Lake, Daihai Lake, Wuliangsuhai Lake, Juyan Lake, Qehan Lake, Wulagai Lake Group, Hongjian Nuur, Bosten lake, Ebi Lake and Jili Lake (see Supplementary Text 3 for more details). As a result, the lake decrease over the plateau was closely correlated with irrigation (*R*^2^ = 0.66, *P* < 0.01) and mining (*R*^2^ = 0.67, *P* < 0.01) but weakly with climate (*R*^2^ = 0.02, *P* > 0.5) (Supplementary Fig. 8).

The Eastern Plain is China’s economy and population center, with the middle and lower reaches of the Yangtze River as its core region. Almost all lakes in this region have been influenced by human activities through dam construction, river regulation and, in particular, lake impoldering—the process of converting lakes into croplands and city lands [4,15–18]. Although lake impoldering in this region has a long history (Supplementary Text 2), the recent decades are exceptional in the severity of this issue. Using land-use maps, we estimated an impoldering area of ∼1100 km^2^ between 1992 and 2015 (Supplementary Fig. 9) and most lakes with low climate influences have suffered from impoldering (see Supplementary Fig. 10 for a map). Existing research also confirmed impoldering as a major driver of lake decrease by calculating the water balance of typical lakes including Dongting Lake, Poyang Lake and Taihu Lake (Supplementary Text 3). At the regional level, similar to the Mong-Xin Plateau, the human factor (i.e. impoldering) emerged as a more significant explanatory variable (*R*^2^ = 0.53, *P* < 0.05) for the lake decrease than climate (*R*^2^ = 0.20, *P* > 0.2) (Supplementary Fig. 11).

## CONCLUSIONS AND PERSPECTIVES

Lakes in China have changed with tremendous regional differences in the past three decades. In the Tibetan Plateau, where lakes were abundant but mostly unavailable to humans, lakes increased because of increased precipitation and warming-induced meltwaters. Conversely, in the densely populated regions, where lake resources are relatively limited but in great demand, lakes decreased further. Although climate change has been generally regarded as the dominant driver of lake changes in China’s history (Supplementary Text 2), the recent lake decreases were largely related to human activities, particularly in the Mong-Xin Plateau and the Eastern Plain. As a result, the regional imbalance between lake resources and population distribution in China is getting worse. Some of the growing lakes in the Tibetan Plateau even pose the risk of outburst flooding [32]. Further exacerbation of these situations is highly likely, given the projected climatic warming and economic growth [33,34]. The governments are undertaking several initiatives to address these lake issues (see Supplementary Text 4 for details). In light of our findings, we suggest that priorities should be given to curb grassland reclamation, lake impoldering, problematic mining and irrigation. Securing a sustainable future for China’s lakes ultimately lies in the wise management of these treasured natural resources and the concerted efforts at the national level.

## Methods

### Landsat images and processing

To monitor the changes in all lakes >1 km^2^ in China, we downloaded a large number of Landsat Thematic Mapper, Enhanced Thematic Mapper Plus (ETM+) and Operational Land Imager images from the USGS website (www.usgs.gov). The spatial resolution of these images is 30 m, and the temporal coverage is from 1985 to 2015/16, with a time-step of 16 days. We used images acquired in the period of June–September when most lakes in China reach their annual maximum area [6]. To ensure the highest quality for lake-area extraction, we visually checked each image and downloaded only those images in which lake boundaries were clearly presented.

The classical normalized difference water index (NDWI) method was then used to delineate the lake boundaries [35,36]. To get the most accurate database of China’s lakes, all artificial reservoirs, ponds and salt lakes without obvious water bodies were excluded by referring to Google Earth images. In this study, artificial reservoirs refer to the water regions created by humans by intercepting rivers during the past three decades. Natural lakes that have been managed by humans during the past 30 years were included in the database because they are direct evidence of human influences. Additional visual inspection and manual correction on each lake boundary were also performed to ensure accuracy.

### Lake changes

We then compiled a database for all lakes larger than 1 km^2^ in China (Supplementary Database) and calculated the changes in the number and area of lakes between the mid-1980s and 2015. Following the approach of previous researches [4,5], we divided the lakes into three size classes to present the lake changes in a clear and informative way: small lakes with areas between 1 and 10 km^2^, medium lakes with areas between 10 and 50 km^2^, and large lakes with areas >50 km^2^.

To better illustrate the regional characteristics of the lakes and following the methods of previous studies [4–6], we divided China’s lakes into five lake regions: the Eastern Plain, the Mong-Xin (Mongolia-Xinjiang) Plateau, the Northeast Plain, the Yun-Gui (Yunnan-Guizhou) Plateau and the Tibetan Plateau lake regions (Fig. 1). The population in each lake area was calculated. Specifically, we used the population of provinces (cities) Jiangsu, Zhejiang, Anhui, Jiangxi, Hubei, Hunan and Shanghai for the Eastern Plain lake region; Inner Mongolia, Xinjiang, Gansu and Ningxia for the Mong-Xin Plateau lake region; Heilongjiang and Jilin for the Northeast Plain lake region; and Yunnan and Guizhou for the Yun-Gui Plateau lake region [37]. The populations of several provinces, such as Henan, Liaoning and Sichuan, were not used because very few natural lakes >1 km^2^ were found there (Fig. 1).

The medium and large lakes in each lake region were then analysed to quantify the temporal changes in lake area (Supplementary Figs 2–6). For this purpose, the RWA of medium and large lakes (>10 km^2^) was calculated for each lake region during nine periods (1985–90, 1991–93, 1994–97, 1998–2000, 2001–03, 2004–07, 2008–10, 2011–13 and 2014–15) using the following equation [7,12]:

$$RWA\left(\%\right)=\displaystyle\frac{1}{n}\sum \limits_{i=1}^n\left({\mathrm{A}}_{\mathrm{i}}/{{\mathrm{A}}_{\mathrm{i}}}^{\mathrm{s}}\right)\times 100$$

where *n*, A_i_ and A_i_^s^ represent the number of the medium and large lakes (>10 km^2^), the area of the *i*th lake and the area of the *i*th lake in the base period, respectively. The base period for each lake region was chosen considering the availability of the images. The RWA method quantifies the overall change trend for a certain region and is free from the influences of lake size. It has been adopted in several pieces of lake-related research [6,19,21]. To check whether seasonal changes in lake area had an influence on the inter-annual trends of lake area, we selected several typical medium and large lakes across China and calculated their inter-annual area using images from both summer and winter seasons (Supplementary Fig. 12). The results indicated that inter-annual trends were similar using images from either summer or winter. Thus, the use of images acquired during summer did not bias the inter-annual trends of lake area.

### Driving forces of lake changes

The driving forces of lake changes were analysed for all medium and large lakes, because they represent a large portion of the total lake area in China. We performed a two-step analysis: first, the strength of climatic control in lake changes was quantified for each medium and large lake. Then, we explored the effects of human activities on lake changes in regions where climate had low influences.

We used annual WA, calculated by subtracting PE from AP, as a potential driver of lake changes for all medium and large lakes. For the Tibetan lakes, we used an additional climate factor—mean annual temperature (MAT)—because it is a good surrogate for meltwater from glaciers, snow and permafrost [25–27]. Temperature was not used for non-Tibetan lakes, because of its overlapping effect with PE and thus with WA. Climate data from ∼ 2400 meteorological stations were obtained from the National Meteorological Information Center of China. We converted PE data from small evaporation pan into E-601B pan using the coefficients provided by Ren *et al.* [38]. We then interpolated climate data into raster layers at 8-km resolution using Anusplin software and extracted climate values over each medium and large lake. Linear model fitting between climate data and lake area was then performed. Analysis of variance (ANOVA) for the linear models was finally conducted to quantify the proportion of variance in lake area explained by climate [6]. This study did not consider groundwater and river flow as controlling factors of lake changes due to data unavailability.

Based on the results of ANOVA, we further analysed the lake changes in the Mong-Xin Plateau and the Eastern Plain lake regions where influences of climate on lake changes were found to be low. Land-use maps and literature review (Supplementary Text 3) identified irrigation and mining as major driving forces of lake changes in these two regions; we thus analysed the effects of irrigation and mining on lakes. For the Mong-Xin Plateau lake region, we first quantified the spatial and temporal patterns of grassland reclamation using annual land-cover maps at 300-m resolution (Annual Global Land Cover Maps v2.0.7; http://cci.esa.int/) (Supplementary Fig. 7). The intensities of cropland irrigation and coal mining were then acquired by referring to statistic yearbooks [28,29] and their influences on lake changes at the regional level were quantified (Supplementary Fig. 8). For the Eastern Plain lake region, we mapped the location and intensity of impoldering using annual land-cover maps (Supplementary Fig. 9). Then, we checked whether lakes with low climate influences have suffered from impoldering (Supplementary Fig. 10). Finally, the impacts of impoldering on regional RWA changes were explored (Supplementary Fig. 11). Direct evidence of irrigation, mining and impoldering on individual lakes was well documented by existing case studies (Supplementary Text 3) and we thus included them in our analyses.

## Supplementary Material

nwz103_Supplemental_Files
