# Supplementary material for: Changes in China’s lakes: climate and human impacts
Source: Natl Sci Rev. 2019 Jul 25;7(1):132–40. doi: 10.1093/nsr/nwz103 (PMC8288840; doi:10.1093/nsr/nwz103)
Supplement: nwz103_Supplemental_Files [file nwz103_supplemental_files.zip › Supplementary Data-NSR_MS-2018-193/Supplementary_Figures_Texts.docx]

**Changes in China’s lakes: Climate and human impacts**

**Supporting Figures S1*–*S12**

**Supplementary Texts 1–4**

**Supporting Figures**


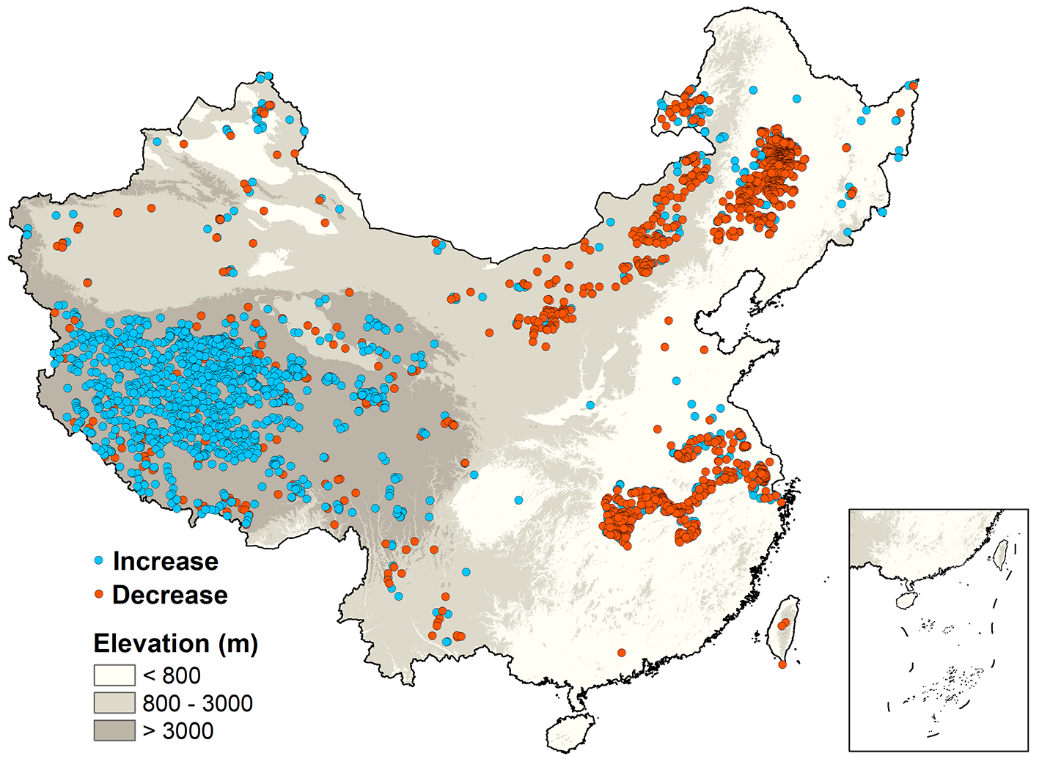


**Fig. S1.** Spatial pattern of lake changes in China between the mid-1980s and 2015.


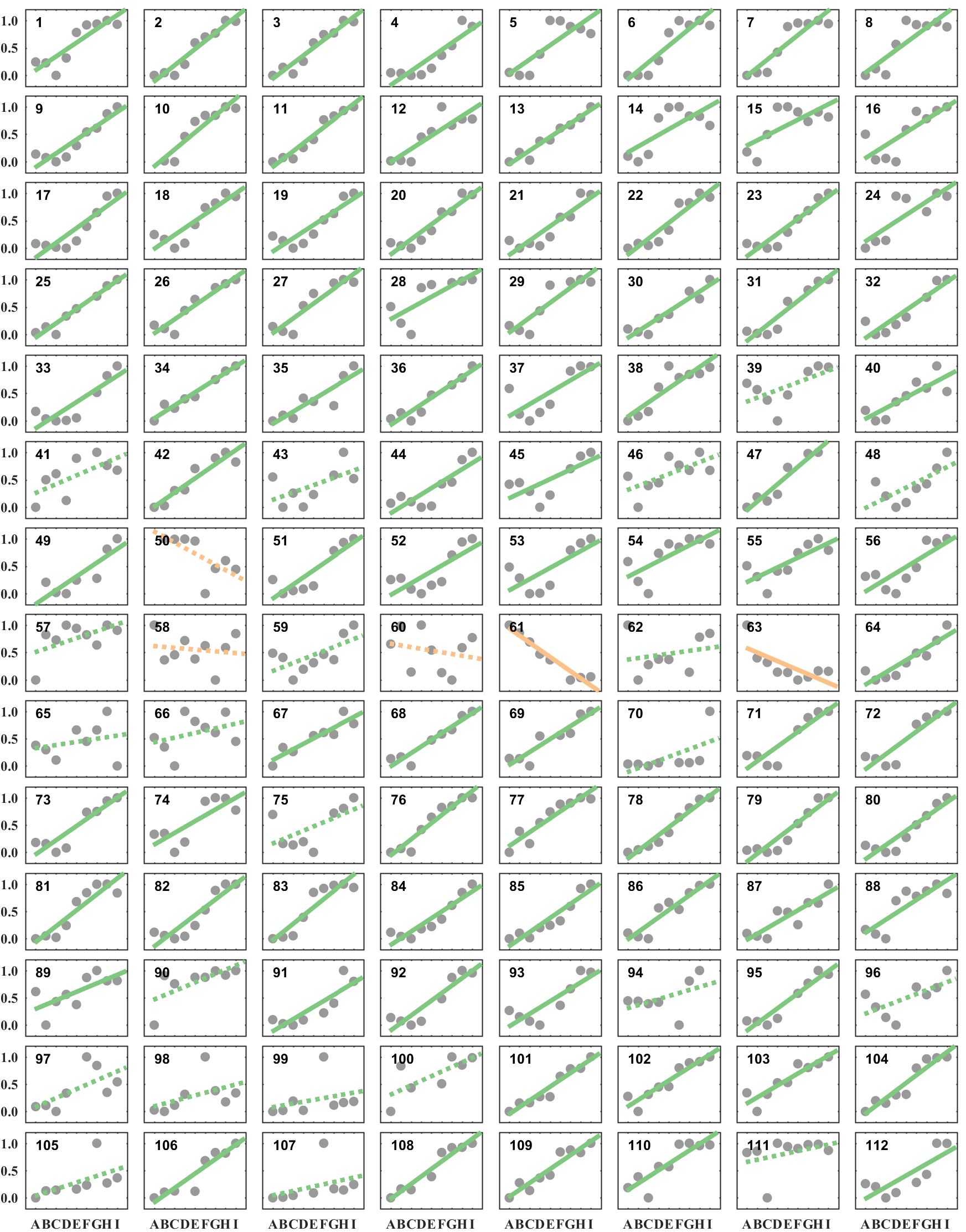


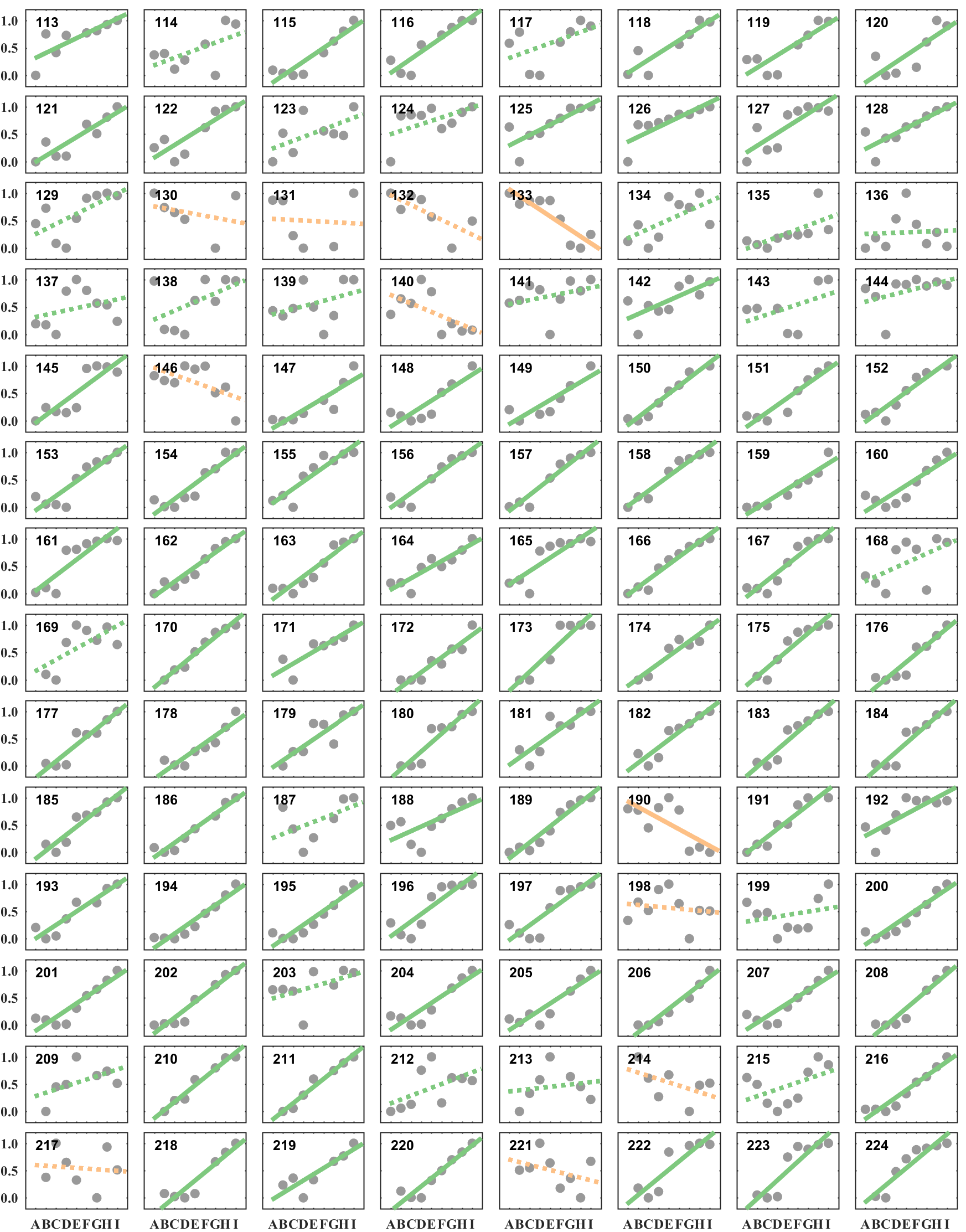

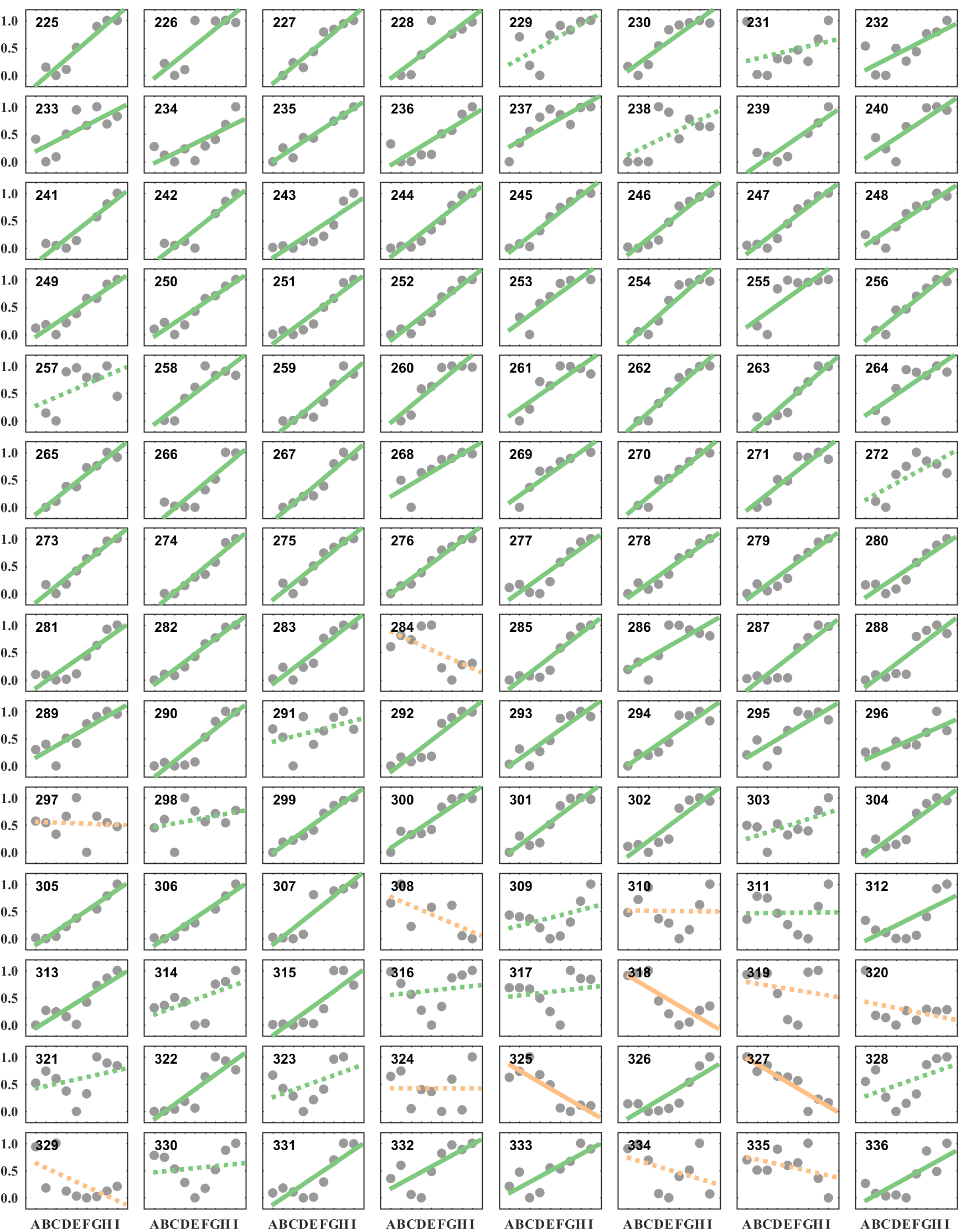

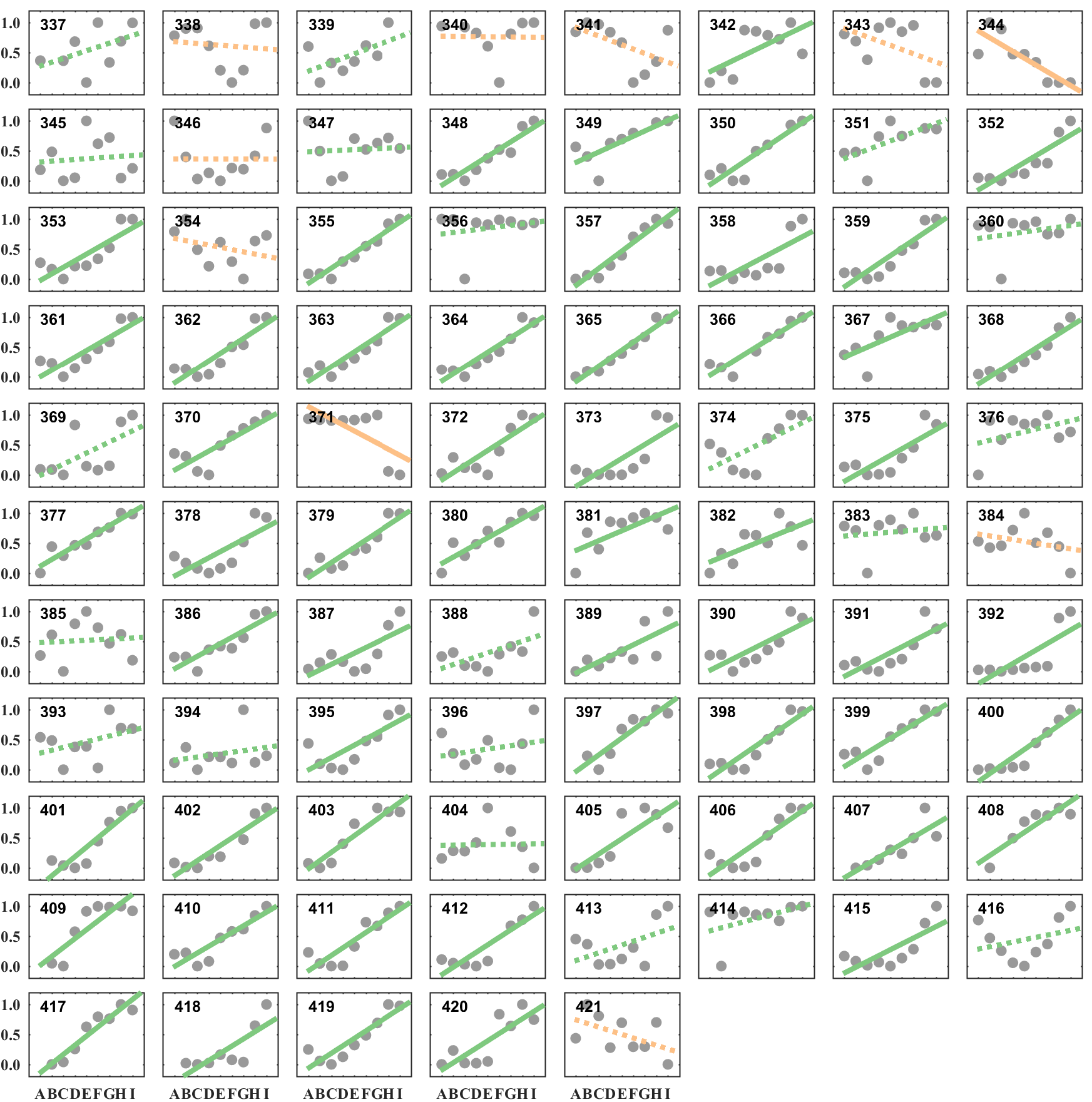


**Fig. S2.** Temporal changes of all the medium and large lakes in the Tibetan Plateau lake region. Green lines represent increasing trends in lake area and yellow lines represent decreasing trends. Solid lines indicate that the trends are significant (*P*<0.05) and dashed lines indicate nonsignificant trends. A–I at *x*-axis indicate 1985–1990, 1991–1993, 1994–1997, 1998–2000, 2001–2003, 2004–2007, 2008–2010, 2011–2013, and 2014–2015, respectively. *Y*-axis has been standardized by setting maximum value as 1. The ID number for each lake is consistent with ChinaLake database.

**
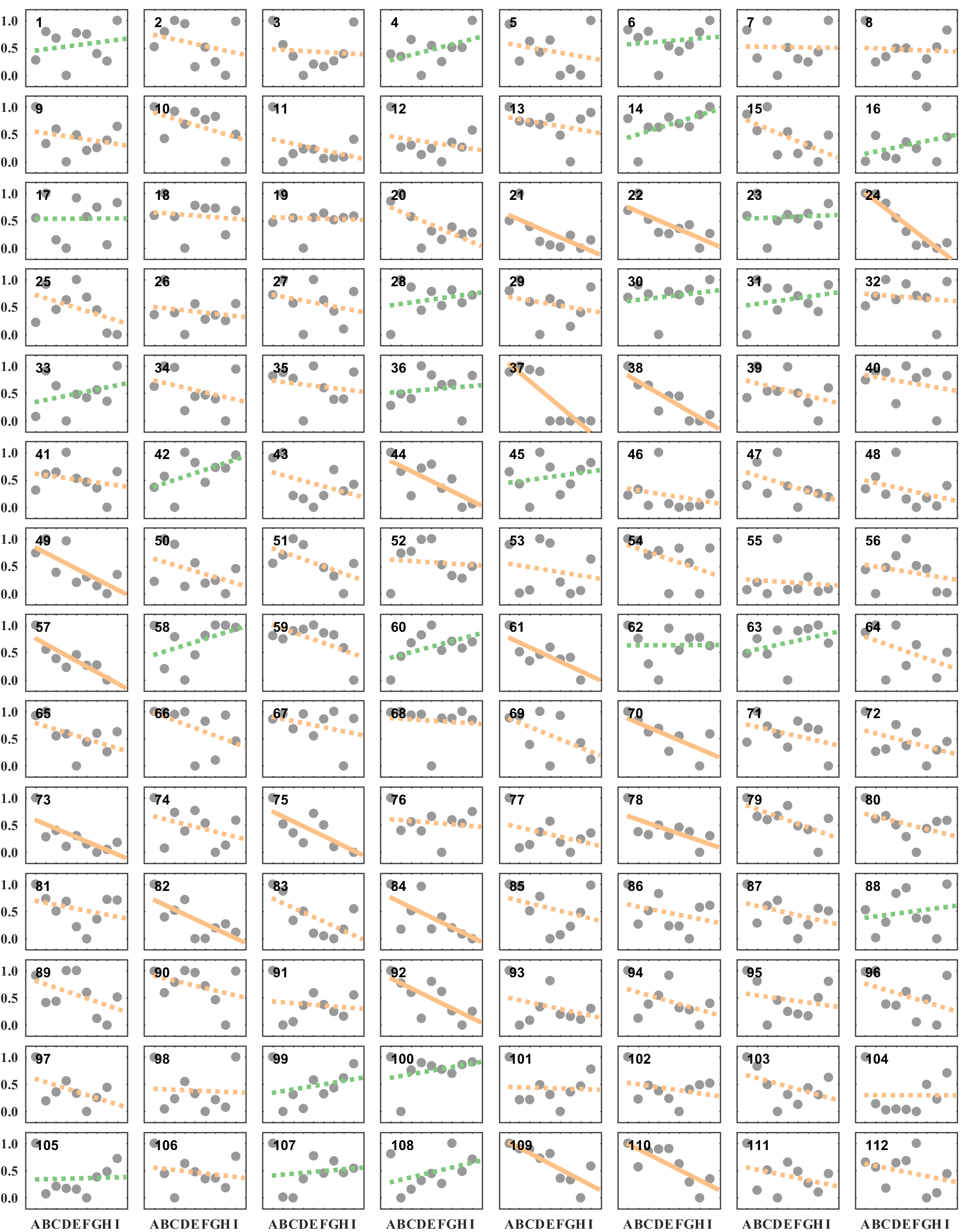

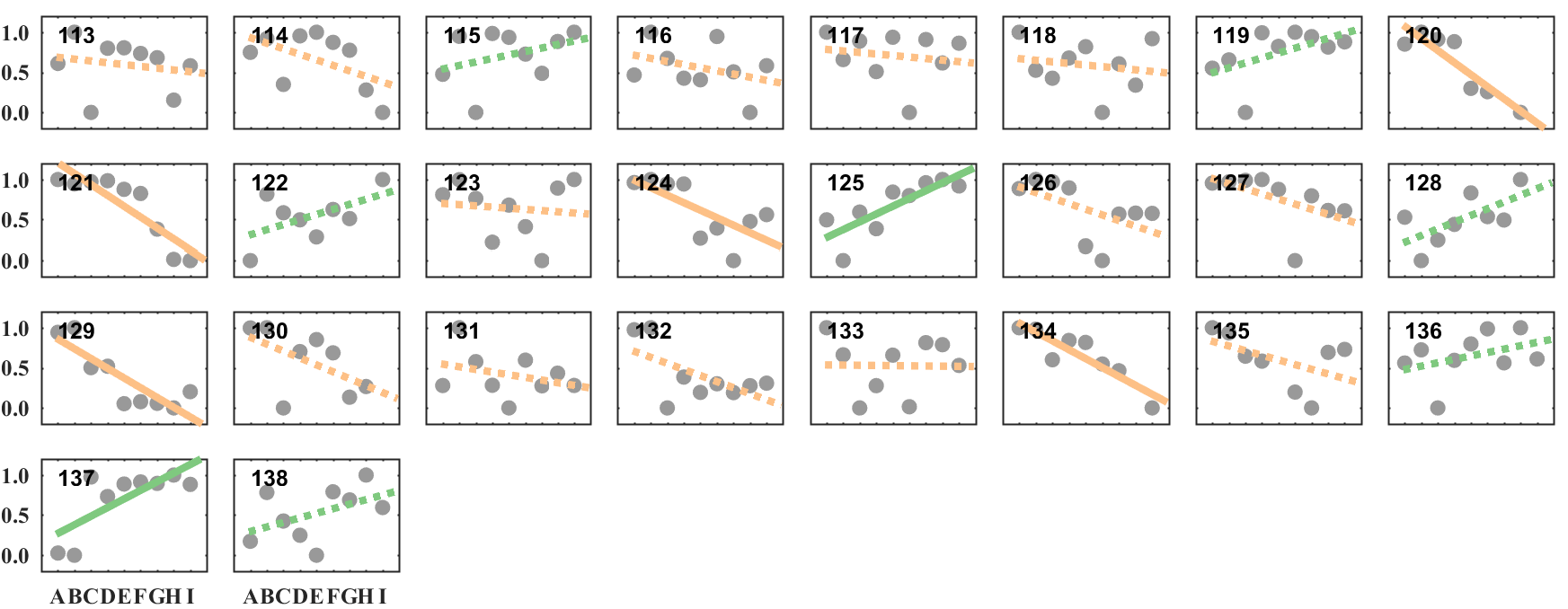
**

**Fig. S3.** Temporal changes of all the medium and large lakes in the Eastern Plain lake region. Green lines represent increasing trends in lake area and yellow lines represent decreasing trends. Solid lines indicate that the trends are significant (*P*<0.05) and dashed lines indicate nonsignificant trends. A–I at *x*-axis indicate 1985–1990, 1991–1993, 1994–1997, 1998–2000, 2001–2003, 2004–2007, 2008–2010, 2011–2013, and 2014–2015, respectively. *Y*-axis has been standardized by setting maximum value as 1. The ID number for each lake is consistent with ChinaLake database.

**
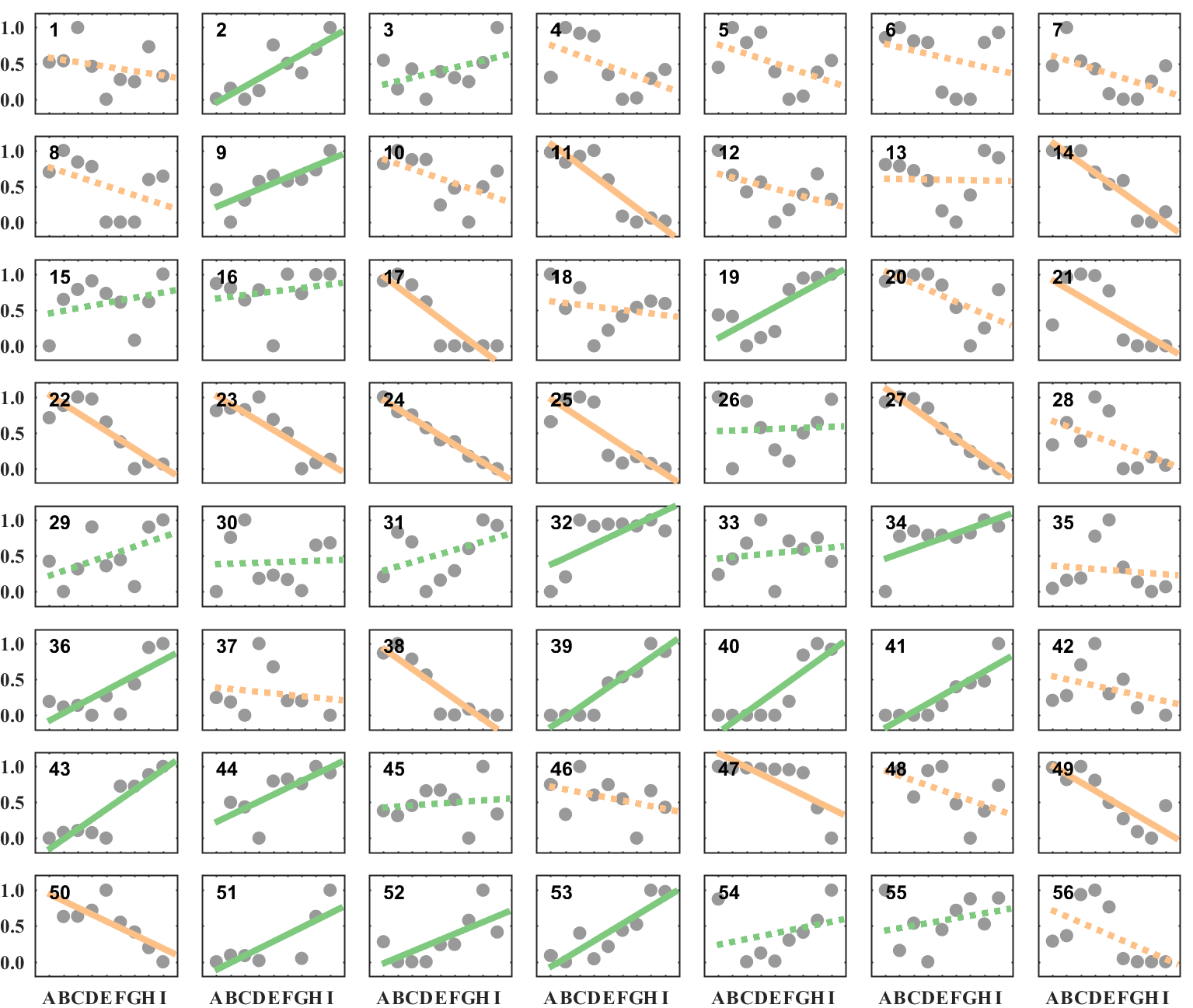
**

**Fig. S4.** Temporal changes of all the medium and large lakes in the Mong-Xin Plateau lake region. Green lines represent increasing trends in lake area and yellow lines represent decreasing trends. Solid lines indicate that the trends are significant (*P*<0.05) and dashed lines indicate nonsignificant trends. A–I at *x*-axis indicate 1985–1990, 1991–1993, 1994–1997, 1998–2000, 2001–2003, 2004–2007, 2008–2010, 2011–2013, and 2014–2015, respectively. *Y*-axis has been standardized by setting maximum value as 1. The ID number for each lake is consistent with ChinaLake database.

**
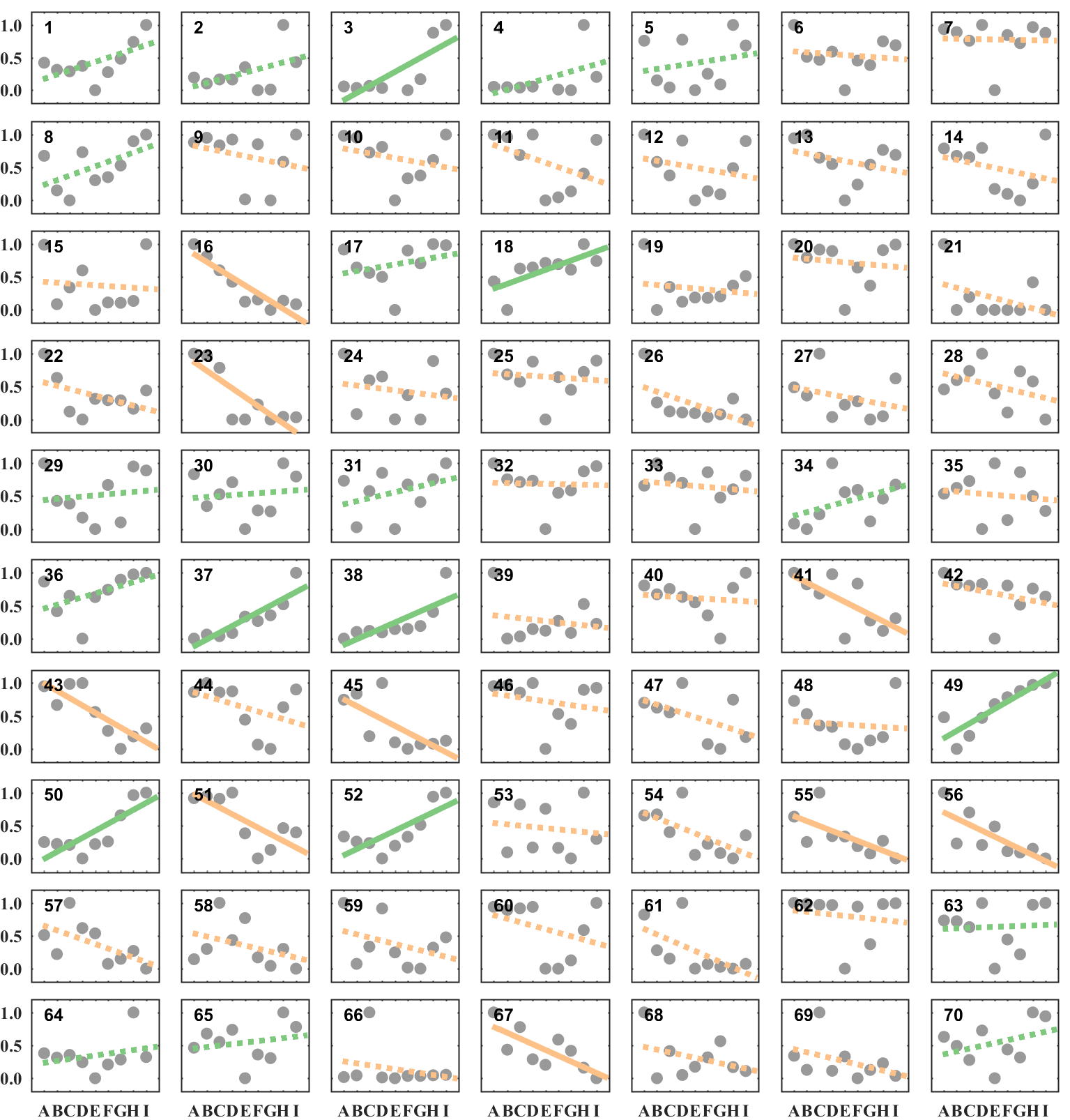
**

**Fig. S5.** Temporal changes of all the medium and large lakes in the Northeast Plain lake region. Green lines represent increasing trends in lake area and yellow lines represent decreasing trends. Solid lines indicate that the trends are significant (*P*<0.05) and dashed lines indicate nonsignificant trends. A–I at *x*-axis indicate 1985–1990, 1991–1993, 1994–1997, 1998–2000, 2001–2003, 2004–2007, 2008–2010, 2011–2013, and 2014–2015, respectively. *Y*-axis has been standardized by setting maximum value as 1. The ID number for each lake is consistent with ChinaLake database.

**
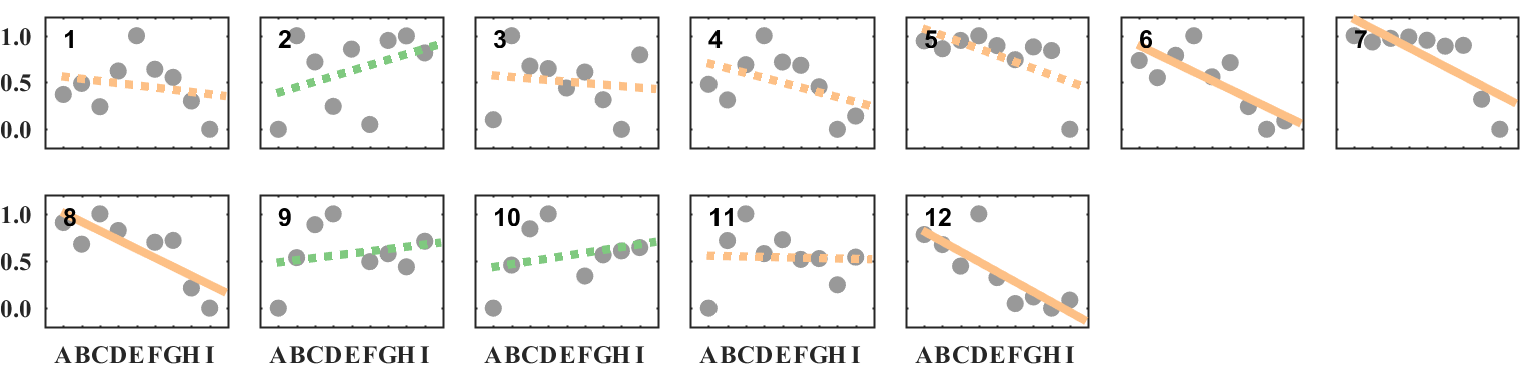
**

**Fig. S6.** Temporal changes of all the medium and large lakes in the Yun-Gui Plateau lake region. Green lines represent increasing trends in lake area and yellow lines represent decreasing trends. Solid lines indicate that the trends are significant (*P*<0.05) and dashed lines indicate nonsignificant trends. A–I at *x*-axis indicate 1985–1990, 1991–1993, 1994–1997, 1998–2000, 2001–2003, 2004–2007, 2008–2010, 2011–2013, and 2014–2015, respectively. *Y*-axis has been standardized by setting maximum value as 1. The ID number for each lake is consistent with ChinaLake database.


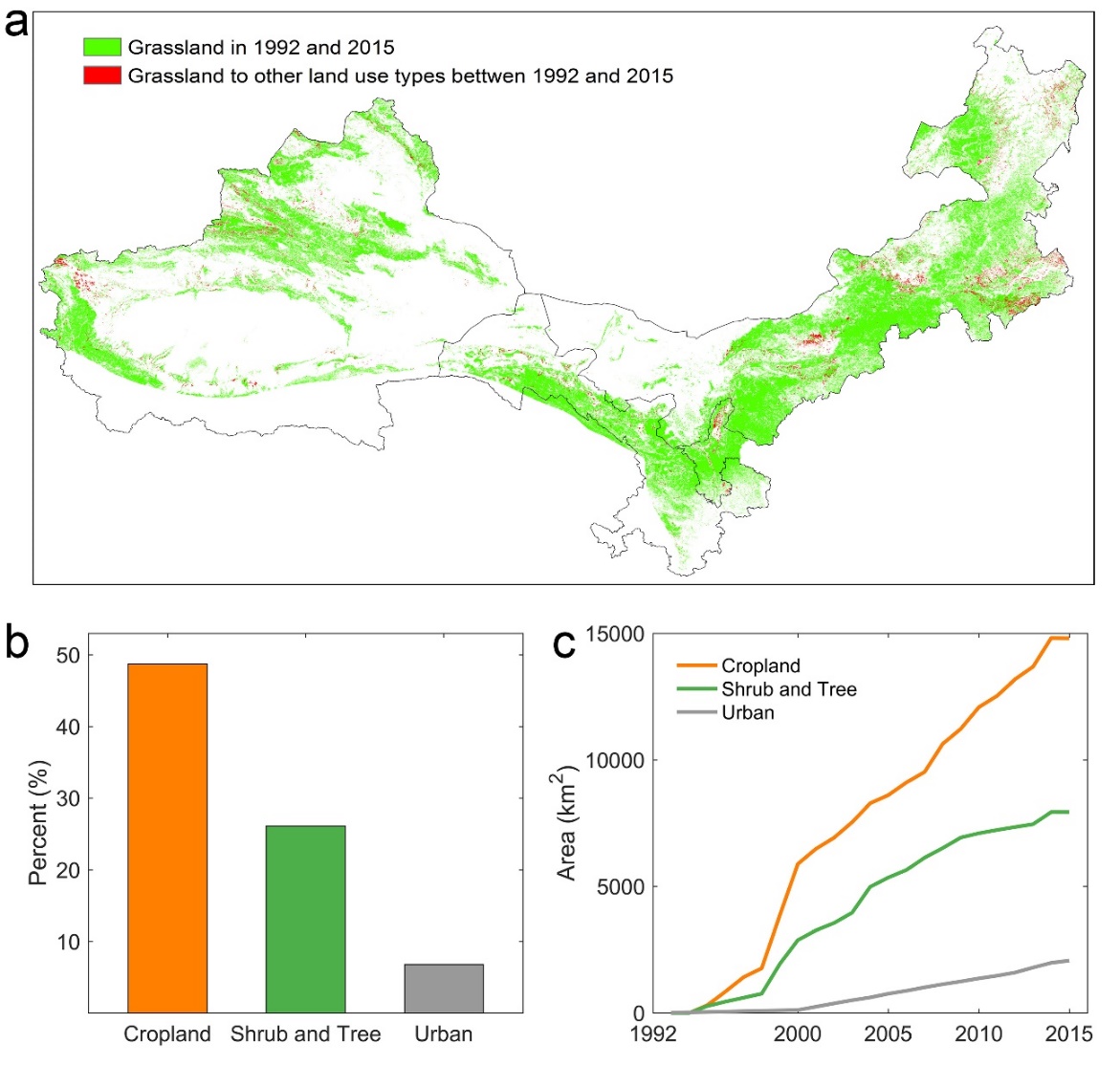


**Fig. S7.** Grassland reclamation in the Mong-Xin Plateau lake region. (a) Spatial pattern, (b) percentages of major land-use types converted from grasslands, and (c) temporal changes of the major land-use types converted from grasslands.


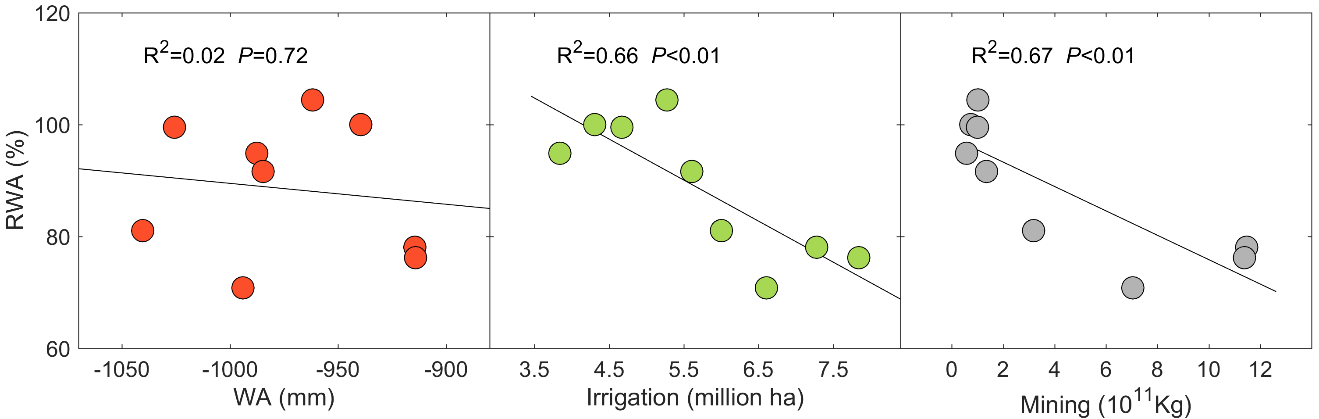


**Fig. S8.** Explanatory powers of climatic water availability (WA, calculated by subtracting annual pan evaporation from annual precipitation), irrigation and mining on lake changes in the Mong-Xin Plateau lake region. RWA means the relative water area (RWA) of all medium and large lakes in the lake region (see Methods). To match RWAs, annual time series of WA, irrigated cropland area, and mining intensity (coal production) were averaged for the nine RWA periods.


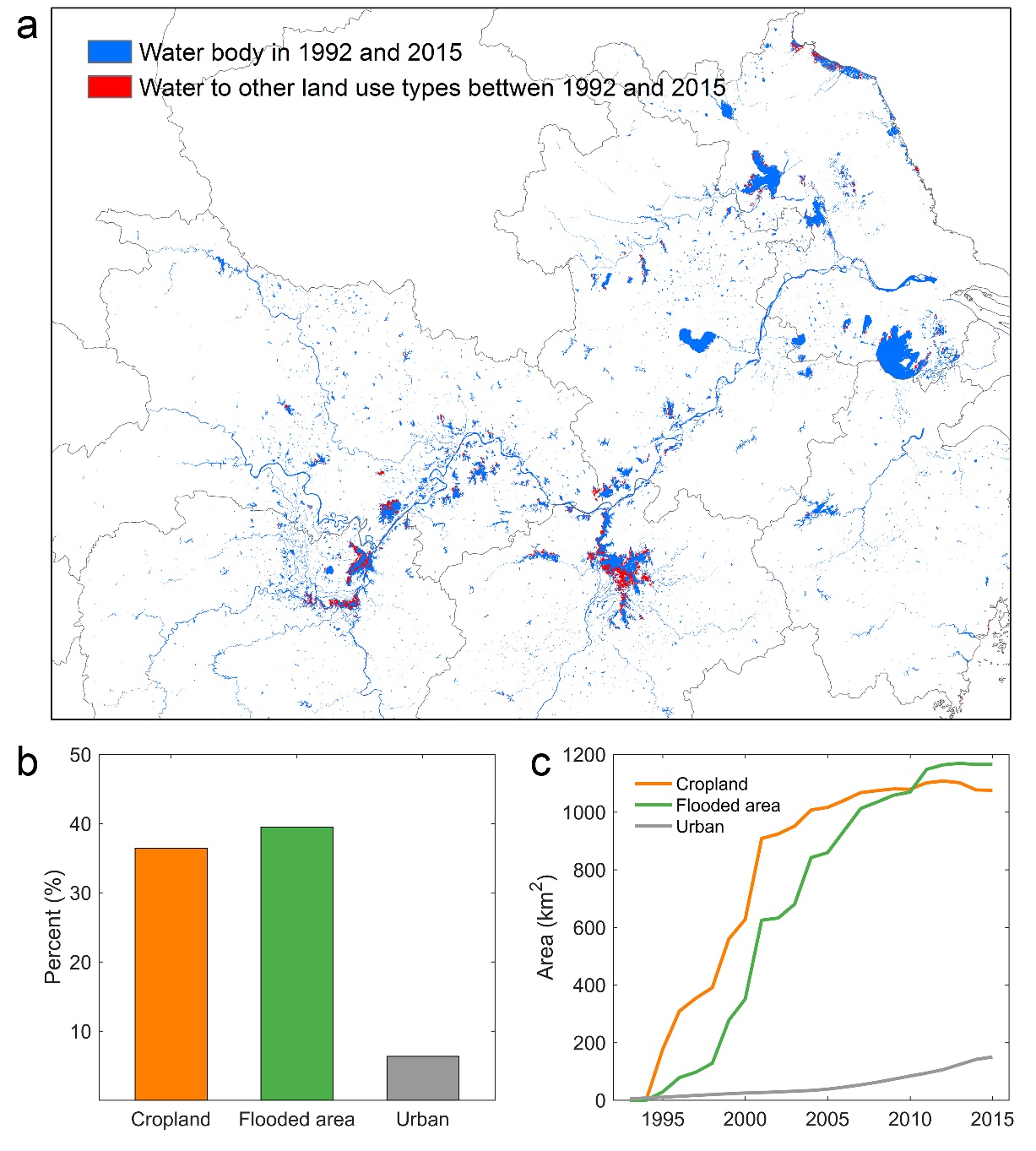


**Fig. S9.** Land-use changes in the middle and lower reaches of the Yangtze River, the core region of the Eastern Plain lake region. (a) Spatial pattern, (b) percentages of major land-use types converted from lakes, and (c) temporal changes of the major land-use types converted from lakes.


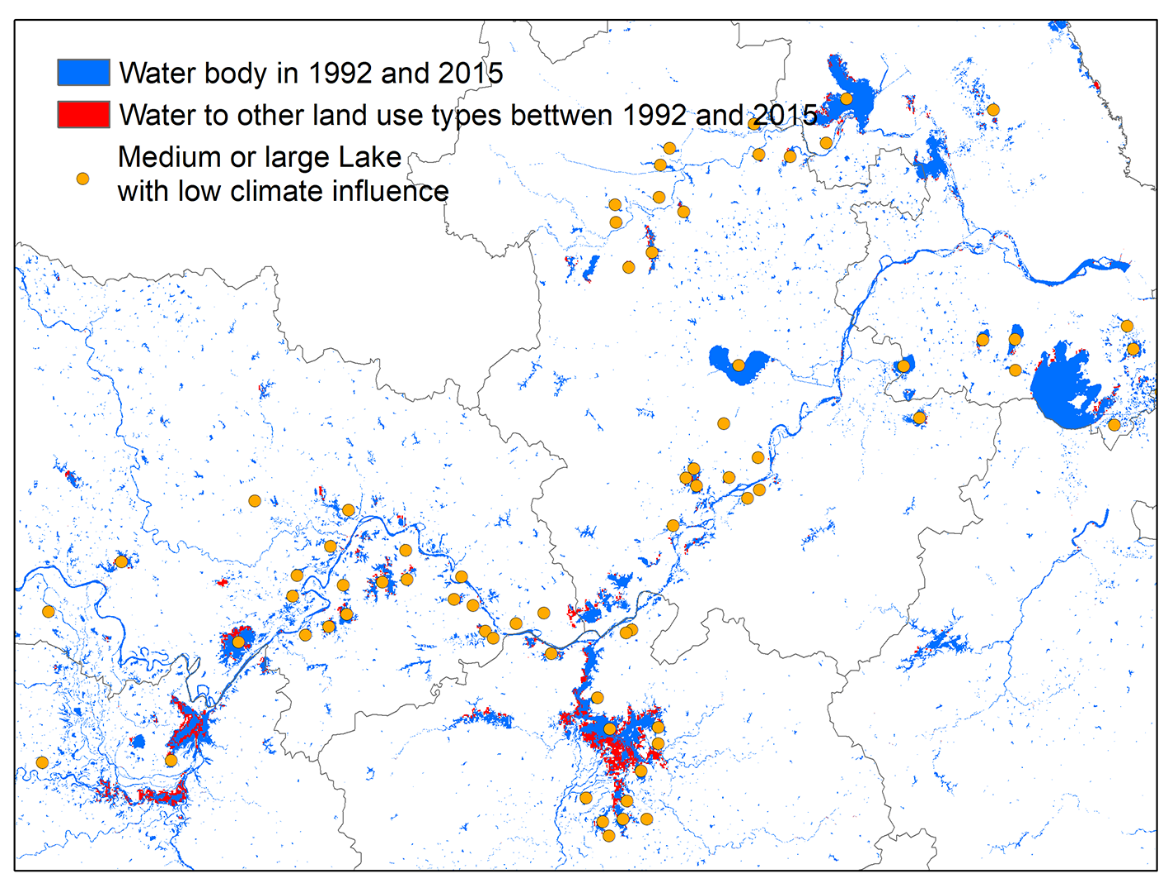


**Fig. S10.** Distribution of lakes with low climate influences against spatial pattern of impoldering in the middle and lower reaches of the Yangtze River (the core area of the Eastern Plain lake region).


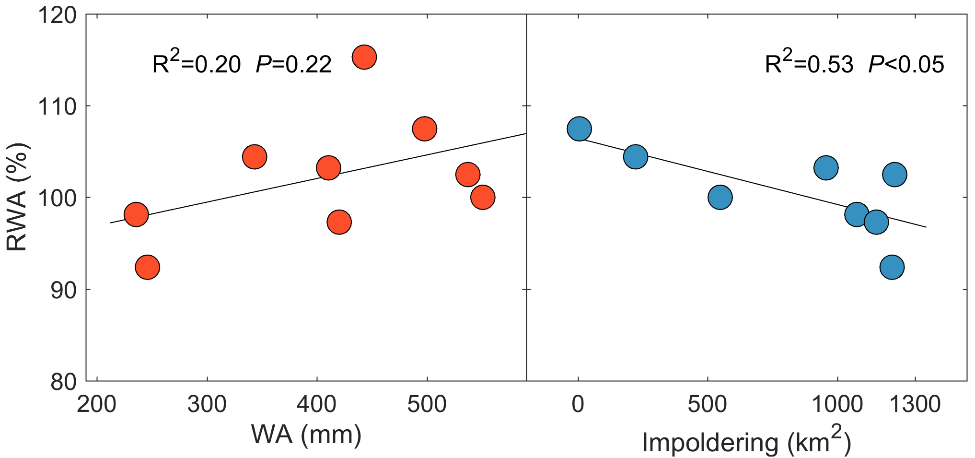


**Fig. S11.** Explanatory powers of climatic water availability (WA, calculated by subtracting annual pan evaporation from annual precipitation) and impoldering on lake changes at reginal level in the middle and lower reaches of the Yangtze River (the core area of the Eastern Plain lake region). RWA means the relative water area (RWA) of all medium and large lakes in the lake region (see Methods). To match RWAs, annual time series of WA and impoldering data were averaged for the nine RWA periods.


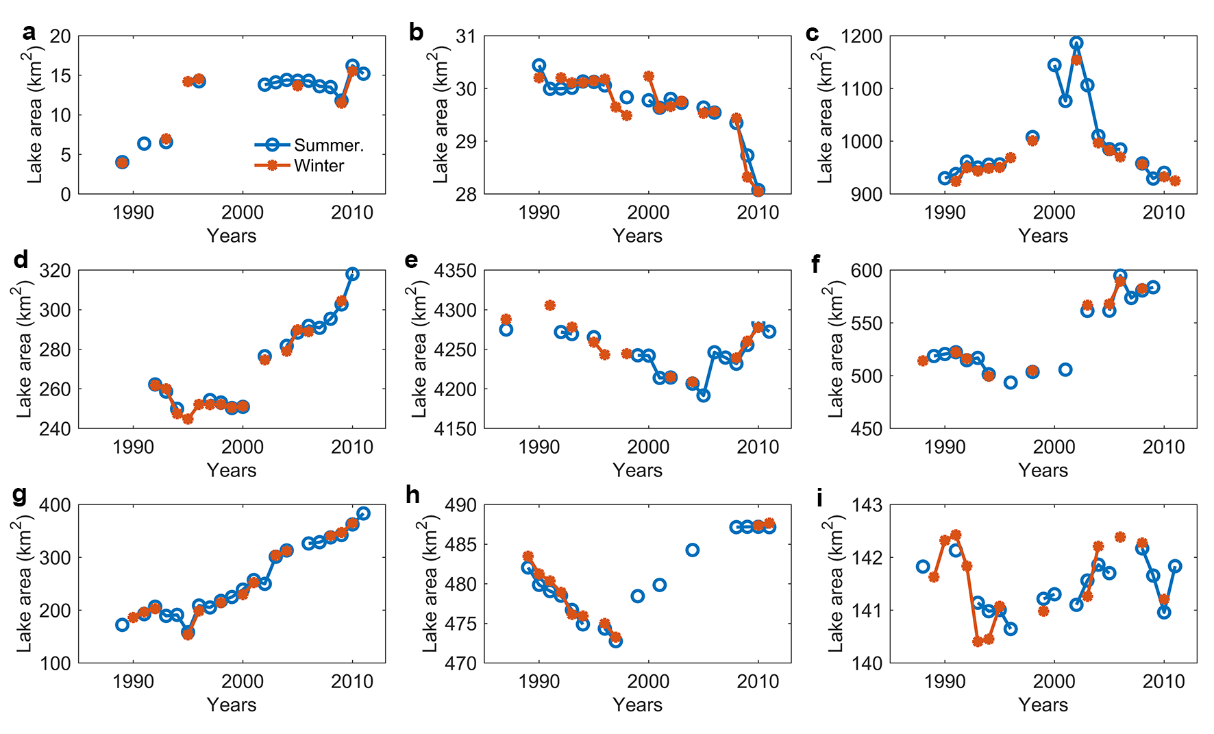


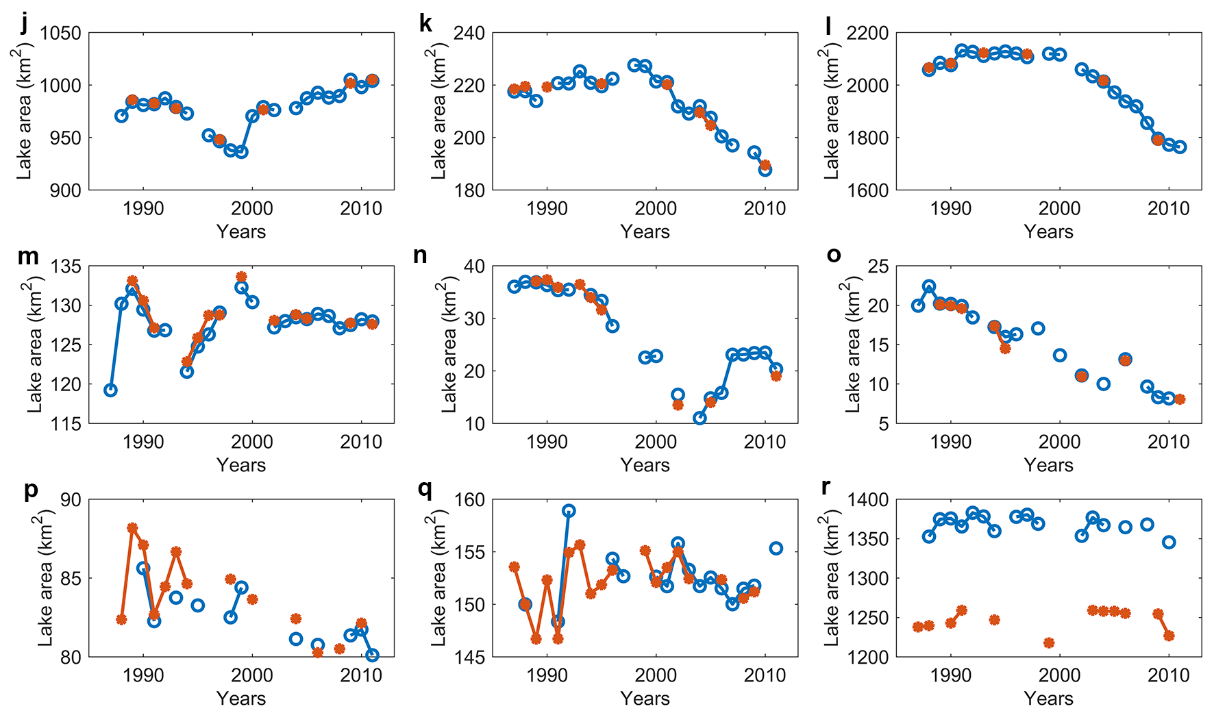


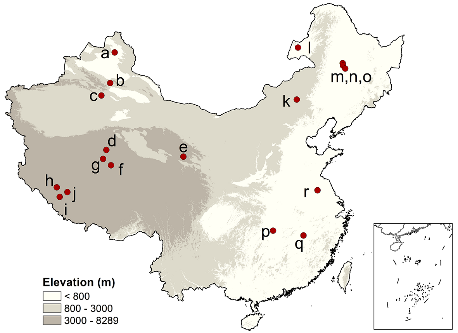


**Fig. S12.** Inter-annual trends of lake area in summer (July–September) and winter (November to January of the following year) for several medium and large lakes in China.

**Supplementary Text 1**

*Descriptions of the five lake regions in China*

***Tibetan Plateau lake region***

Located at the vast elevated plateau in western China, the Tibetan Plateau lake region is the largest lake region in China and also the highest lake region of the world. It covers Tibet Autonomous Region and Qinghai Province, as well as part of Xinjiang Uyghur Autonomous Region, Sichuan- and Yunnan- Province. Most of the lakes in this region are fed by meltwater from glaciers and permafrost, and precipitation. Because of the unique geographic and climatic features of Tibetan Plateau, many lakes are not impacted by human activities and deemed sacred by local people (*1*). The most famous lakes in this region include, to name a few, Nam Co, the highest saline lake in the world, and Qinghai Lake, the largest lake in China (~4275 km^2^ in the mid-1980s). According to our calculation, there were 1065 lakes with water area > 1 km^2^ in the mid-1980s in the Tibetan Plateau (i.e., 38.1% of the total number of lakes in China), and their total lake area was 38,596 km^2^ (51.3% of the total lake surface area in China). Lake number and lake area for all lake-size classes ranked the first in China (Table 1 in the main text). Specifically, the number of small (1–10 km^2^), medium (10–50 km^2^), and large (>50 km^2^) lakes was 654, 228, and 183, respectively, and their area was 1,543, 3,645, and 33,408 km^2^, respectively (Fig. 1 and Table 1 in the main text).

***Eastern Plain lake region***

As the second largest lake region in China, the Eastern Plain lake region spans across the vast east plains of China, with the middle and lower reaches of the Yangtze River as its core region. There were 621 lakes with water area > 1 km^2^ in the mid-1980s in this region (22.2% of the total number of lakes in China), and their total lake area was 19,384 km^2^ (25.8% of the total lake area in China). The number of small, medium, and large lakes was 483, 89, and 49, respectively, and their area was 1,573, 1,917, and 15,895 km^2^, respectively (Fig. 1 and Table 1 in the main text). Lakes in this region are mainly fed by precipitation and rivers, and many of them are connected to the Yangtze River. Together with the Yangtze River, lakes in this region function as one of the most species-rich wetlands (the last refuge of *Lipotes vexillifer* and *Alligator sinensis*), the earliest rice-growing regions, and the busiest shipping arteries in the world (*2–5*). Moreover, all the five largest freshwater lakes in China, namely Poyang Lake in Jiangxi Province, Dongting Lake in Hu’nan Province, Tai- and Hongze- Lake in Jiangsu Province, and Chao Lake in Anhui Province, are located in this region. The importance of these large lakes to China’s agriculture and even culture is pronounced since ancient China. For example, Dongting Lake is the birthplace of dragon boat racing; the regions around Tai Lake has been traditionally called the “country of rice and fish” (*6*).

***Mong-Xin Plateau lake region***

Located in the vast arid and semi-arid lands in East Asia, the Mong-Xin (Mongolia-Xinjiang) Plateau lake region is the third largest lake region in China. It is also a major part of Eurasia steppe and the Mongolian Plateau. There were 531 lakes with water area > 1 km^2^ in the mid-1980s in this region (19.0% of total lake number in China), and their total lake area was 8,173 km^2^ (10.9% of total lake area in China). The number of small, medium, and large lakes was 478, 30, and 23, respectively, and their area was 1,260, 463, and 6,450 km^2^, respectively (Fig. 1 and Table 1 in the main text). Water resource in the Mong-Xin lake region is therefore extremely scarce and lakes are a valuable water source for grasslands, irrigated agriculture, industry, and many endangered species. Some saline lakes also provide economic products such as mirabilite. Except for a few lakes in Xinjiang which receive meltwater from glacier and snow, most lakes in the plateau are fed by groundwater, rivers and precipitation. It is worth mentioning that, in addition to the lakes in the vast grasslands, there are a few inter-dune lakes distributed in the Badain Jaran Desert in Inner Mongolia (*7*) —a quite unique natural scenery that attracts both scientists and tourists. However, during the past decades, lakes in the plateau were under tremendous pressures from irrigation and industry activities such as mining (*8, 9*).

***Northeast Plain lake region***

This lake region spans across three provinces in northeast China (Jilin, Heilongjiang and Liaoning Province), with Songnen Plain in Jilin and Heilongjiang Province as its core region. We identified 555 lakes with water area > 1 km^2^ in the mid-1980s in this region (19.8% of total lake number in China), and their total lake area was 8,045 km^2^ (10.7% of total lake area in China). The number of small, medium, and large lakes was 485, 56, and 14, respectively, and their area was 1,252, 949, and 5,845 km^2^, respectively (Fig. 1 and Table 1 in the main text). Lakes in this region are mainly fed by rivers and precipitation. There are a few lava-dam lakes and crater lakes in this region, such as Tianchi Lake in the Changbai Mountains – the deepest lake in China (*10*) (maximum depth of 384 m). Agricultural activities such as irrigation are increasingly influencing the lakes in this region (*11*).

***Yun-Gui Plateau lake region***

The Yun-Gui (Yunnan-Guizhou) Plateau lake region is adjacent to the Tibetan Plateau, with Yunnan and Guizhou Province as its core region. Lake number and area were both the least in China: there were only 27 natural lakes with water area > 1 km^2^ in the mid-1980s (1% of total lake number in China), with a total lake area of 1057 km^2^ (1.4% of total lake area in China). The number of small, medium, and large lakes was 15, 8, and 4, respectively, and their area was 46, 192, and 819 km^2^, respectively (Fig. 1 and Table 1 in the main text). Despite their small numbers, lakes in this region are among the most popular tourist attractions in China.

References

1. Salick, J. *et al*. Tibetan sacred sites conserve old growth trees and cover in the eastern Himalayas. *Biodivers Conserv* **16**, 693 (2007).
2. Xie, P., Chen, Y.Y. Threats to biodiversity in Chinese inland waters. *Ambio* **28**, 674–681 (1999).
3. Fang, J.Y., Rao, S., Zhao, S.Q. Human‐induced long‐term changes in the lakes of the Jianghan Plain, Central Yangtze. *Front Ecol Environ* **3**, 186–192 (2005).
4. Fang, J.Y. *et al*. Biodiversity changes in the lakes of the Central Yangtze. *Front Ecol Environ* **4**, 369–377 (2006).
5. Normile, D. Yangtze seen as earliest rice site. *Science* **275**, 309–309 (1997).
6. Wang, S.M., Dou, H.S. *Lakes in China* (Science Press, Beijing, 1998). [In Chinese]
7. Yang, X.P. *et al*. Recharge to the inter-dune lakes and Holocene climatic changes in the Badain Jaran Desert, western China. *Quat Res* **73**, 10–19 (2010).
8. Tao, S. L. *et al*. Rapid loss of lakes on the Mongolian Plateau. *Proc Natl Acad Sci USA* **112**, 2281–2286 (2015).
9. Fang, L.Q., Tao, S.L., Zhu, J.L., Liu, Y. Impacts of climate change and irrigation on lakes in arid northwest China. *J Arid Environ* **154**, 34–39 (2018).
10. Wei, H., Liu, G., Gill, J. Review of eruptive activity at Tianchi volcano, Changbaishan, northeast China: implications for possible future eruptions. *Bull Volcanol* **75**, 706–719 (2013).
11. Yang, X. *et al*. Adaptation of agriculture to warming in Northeast China. *Clim Change* **84**, 45–58 (2007).

**Supplementary Text 2**

*A short review on the historic changes in China’s lakes*

The historic changes in China’s lakes have been studied by previous researches (*1*–*4*). We reviewed existing researches and found that the historic change in China’s lakes have been a gradual process (time scales of 10^2^–10^6^ years) mainly driven by climate changes.

Between the Last Glacial Maximum and the advent of human civilization, lakes in China changed with climatic changes and tectonic movements at the time scale of 10^3^–10^6^ years (*2*). For the Tibetan Plateau lake region, a radio-carbon chronology showed that the Plateau was in the “great lake periods” in 40~20 ka B.P. (*5*). In the late glacial period (15~12 ka B.P.), increased precipitation and glacier melt, caused by warm and humid climate, enlarged the Tibetan lakes. However, from 11.5 to 10 ka B.P., lakes decreased rapidly in the plateau in response to the Younger Dryas (*6*). In the mid-Holocene (7.5~3 ka B.P.), lakes in the plateau started to increase again due to the high temperatures (*1*). Similarly, the Eastern Plain lake region was also highly responsive to climate changes. In 7.5~3.0 ka B.P. when precipitation was adequate in the eastern China, the lakes increased in size pervasively. At that time, several great lakes formed in the middle reach of the Yangtze River, such as the Yun-meng Ze Lake (cloud-dream swamp; *7*). Starting from 3 ka B.P., the eastern China entered a period of dry and cold conditions, and the great lakes gradually decreased into several small lakes, such as the famous Baiyangdian Lake (*2*). The Mong-Xin Plateau lake region was located within the influencing zones of the East Asia monsoon, and the lakes have been changing with the amount of precipitation brought by the monsoon. In the last glacial maximum with a weak monsoon, lakes in the Mong-Xin Plateau were pervasively small. With the intensification of the monsoon in the mid-Holocene, the lakes increased in area tremendously across the plateau. This “large lake period” was followed by lake shrinkages in the late Holocene (*1*). Lakes in the Yun-Gui Plateau lake region have been changing with the precipitation brought by the southwestern monsoon. For example, lakes in the Yun-Gui Plateau were large in size during 40~20 ka B.P. when monsoon was strong. During 4~2.7 ka B.P., however, the monsoon weakened, and the lakes decreased in size consequently (*8*).

In the era of human civilization, lakes in China have been changing at the time scale of 10^2^ –10^3^ years (*4*), influenced not only by climate changes but also human activities. Since ancient times, two types of human activities have been affecting the lakes in China: impoldering–a process of converting lakes to farmlands or other land-use types, and its opposite, that is returning farmlands to lakes. The peak intensities of impoldering occurred in the Wei-Jin Dynasty, the Song Dynasty and the late Ming to the early Qing period (corresponding to The Little Ice Age subsequent to the 17th century; *2, 3*). In Song Dynasty, more than 80% of the lakes in the East China Plain suffered from impoldering (*3*). The peak intensities of returning farmlands to lakes occurred in the Han Dynasty, the Tang Dynasty and the late Yuan to the early Ming period (*3,4,9*). The earliest impoldering activity in China occurred around Taihu Lake at the lower reach of the Yangtze River in the 11th century B.C. (*1*).

However, researchers argued that the influences of human activities on China’s lakes, even at their peak intensities, were smaller than those of climate changes (*3*). The three peak periods of impoldering were coincided with the three extremely dry periods in ancient China. The dry climate was unfavorable for agriculture, and it also reduced the size of lakes. The lake retreat regions, nutrient-rich with high water content, were then impoldered into farmlands to increase agricultural production (*1*). The three peak periods of returning farmlands to lakes were coincided with the extremely wet periods in China’s history. Massive precipitation forced ancient Chinese to abandon their farmlands, which then became a part of the lakes.

References

1. Wang, S.M., Dou, H.S. Lakes in China (Science Press, Beijing, 1998). [In Chinese]
2. Shen, J. Spatiotemporal variations of Chinese lakes and their driving mechanisms since the Last Glacial Maximum: A review and synthesis of lacustrine sediment archives. *Chini Sci Bull* **58**, 17–31 (2013).
3. Fang, J.Q. Relationships between lake evolution and land reclamation in history of China. *Geogr* *Environ Res* (1), 71–78 (1989). [In Chinese]
4. Fang, J.Q. Lake Evolution during the Last 3000 Years in China and Its Implications for Environmental-Change. *Quatern Res* **39**, 175–185 (1993).
5. Lehmkuhl, F., Haselein, F. Quaternary paleoenvironmental change on the Tibetan Plateau and adjacent areas (Western China and Western Mongolia). *Quatern Int* ***65–6****, 121*(2000).
6. Gu, Z.Y. *et al*. Changes in the monsoon in Tibetan Plateau over the past 12000 years—evidence from Siling Lake. *Chini Sci Bull* **38**, 61–64(1993).
7. Zhou, F. Q. Historical evolution of Yunmeng marsh and Jingjiang delta. *J Lake Sci* **1**, 003 (1994). [In Chinese]
8. Sun, X. J., Wu, Y.S. Holocene vegetation history and environmental changes of the Dianchi lake area, Yunnan Province [in Chinese]. In: Article Collection of China-Australia Quaternary Symposium. Beijing: Science Press, 1987.
9. Chen, Q.Y., Lu,Y.C., Yue, Z.M. On the lacustrine fluctuation in the regions of Ningbo-Shaoning Plain in historical periods. *Geophys Res* **3**, 29–43 (1984). [In Chinese]

**Supplementary Text 3**

*A brief review on existing researches on China’s lake changes*

We conducted a brief review on China’s lake changes. Typical lakes in five lake regions were reviewed (Fig. T3-1). For each lake, we showed its temporal change in lake area, and then illustrated the change reasons by referring to existing case studies.


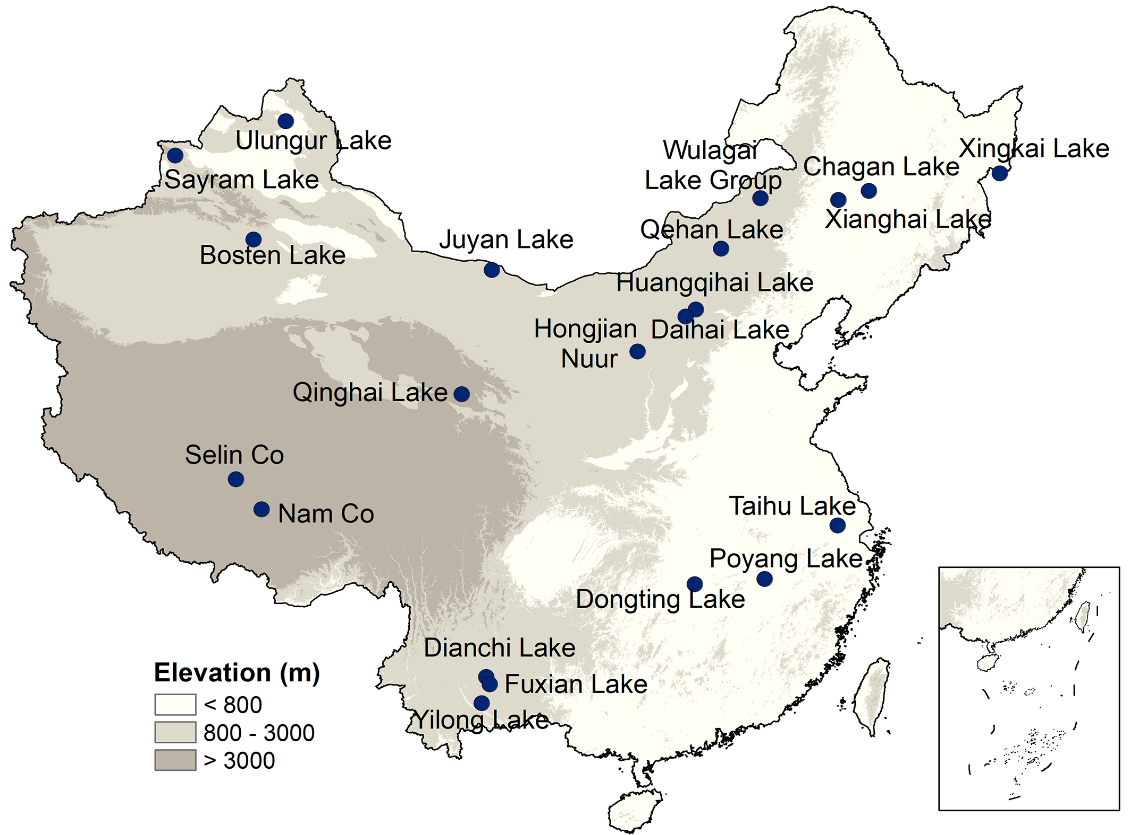


Fig. T3-1. Locations of the lakes reviewed below.

***Tibetan Plateau lake region***

The Tibetan Plateau has abundant lake resources but a very small population. Lakes in the Tibetan Plateau have been changing with climate changes. Existing researches generally agreed that both the warming temperatures and the increasing precipitation contributed to the expansion of Tibetan lakes. We provided three typical examples here.

*Qinghai Lake*

Qinghai Lake, or Tso Ngonpo, is the largest lake in China. It is located in Qinghai Province, the northeast of the Tibetan Plateau. From the mid-1980s to the 2000s, the area of Qinghai Lake exhibited an overall decreasing trend (Fig. T3-2). According to existing researches, the loss of water of Qinghai Lake was greater than the water supply during this period (*1, 2*). Between 1959 and 2000, mean annual precipitation at Qinghai Lake was ~357 mm, surface runoff was ~348 mm, underground runoff was ~138 mm, and evaporation was ~924 mm, which resulted in an annual water level change of about –80 mm (*1*). Similar water balance was observed between 1971 and 2005 (*2*).


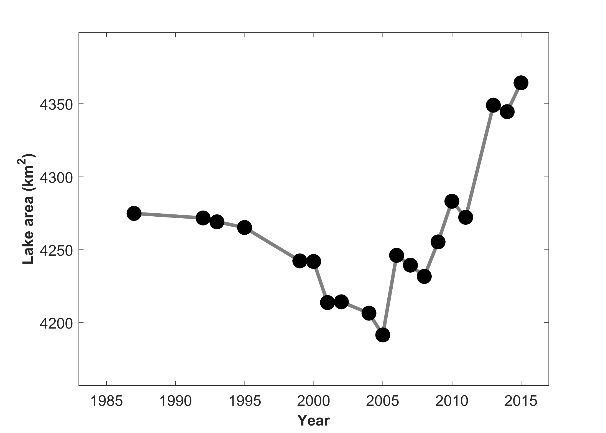


Fig. T3-2. Changes in lake area of Qinghai Lake between the mid-1980s and 2015.

Since the year of 2000, however, the climate in the Qinghai Lake Basin has become warmer and wetter, providing meltwater from glaciers and snow for Qinghai Lake. Since 2004, the precipitation at Qinghai Lake has increased significantly (*3–5*). Consequently, the lake area continued to increase (Fig. T3-2). Besides, the increased lake area was also related to artificial activities such as returning grazing to grassland, returning farmland to forest, and artificial rainmaking (*5*).

*Selin Co*

Selin Co, the largest lake in Tibet, is located in the southern Qiangtang Plateau, Tibet of China. Selin Co receives glacier meltwater through rivers as main water supply. It loses water mainly through soil evaporation and lake surface evaporation (*6, 7*).


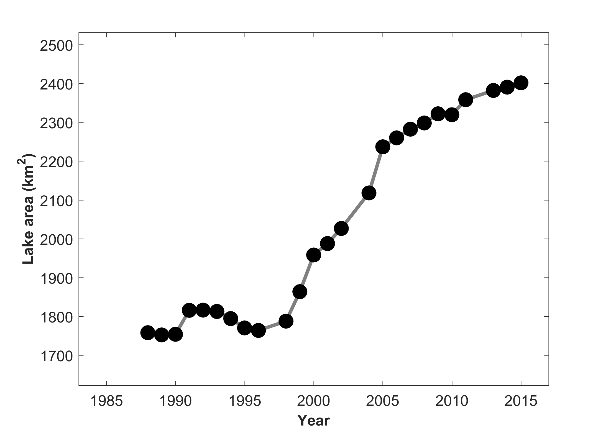


Fig. T3-3. Changes in lake area of Selin Co between the mid-1980s and 2015.

In the past 30 years, the area of Selin Co increased rapidly (Fig. T3-3). The increase of glacier meltwater created by the elevated temperature (0.38 ^o^C/10a, 1970–2016) was deemed as the main reason of the observed lake expansion (*8*). During 1986–2016, the glacier area around Selin Co decreased by 50.16 km^2^ (–7.5%). Additionally, the increase in precipitation (13.72mm/10a, 1970–2016) and the decrease in evaporation (–61.7mm/10a, 1971–2006) also contributed to the expansion of Selin Co (*6, 7*).

*Nam Co*

Nam Co is the second largest lake in Tibet. It has been supplied by rainfall that falls directly on the lake surface, by river runoff created by rainfall, and by glacial meltwater. It loses water mainly through evaporation from the lake surface (*9, 10*).


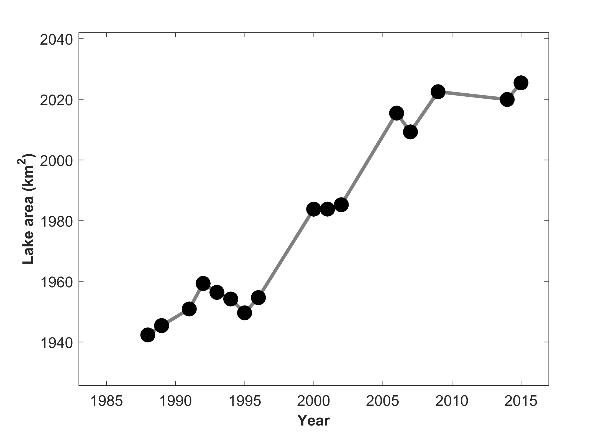


Fig. T3-4. Changes in lake area of Nam Co between the mid-1980s and 2015.

In the last three decades, similar with Selin Co, the area of Nam Co showed a continuous increasing trend (Fig. T3-4). Existing researches concluded that the expansion of Nam Co was mainly caused by the increasing glacial meltwater and precipitation, and the decreasing evaporation (*9, 10*).

***Yun-Gui Plateau lake region***

According to existing researches, lakes in the Yun-Gui Plateau have been changing with climate changes. Especially, the drought occurred during 2009–2012 in the plateau had a large impact on the lakes. Human activities were also reported as a contributor to the lake changes.

*Dianchi Lake*

Dianchi Lake is a famous plateau freshwater lake located in the southwestern part of Kunming City in Yunnan Province. Dianchi Lake has been supplied mainly by rainfall (including the surface runoff created by rainfall), which accounts for ~73% of the total inflow (*11*). Dianchi Lake loses water mainly via evaporation, natural outlet (namely, overflow or outflow), and human consumption. The mean annual precipitation at Dianchi Lake is ~900 mm, and the annual evaporation is ~1,400 mm. Dianchi Lake also provides a large amount of water to Kunming City. It was estimated that the annual water consumption in the Dianchi Lake Basin was ~370 million m^3^, of which ~225 million m^3^ was for domestic water use and ~145 million m^3^ for industrial water use (*11*).


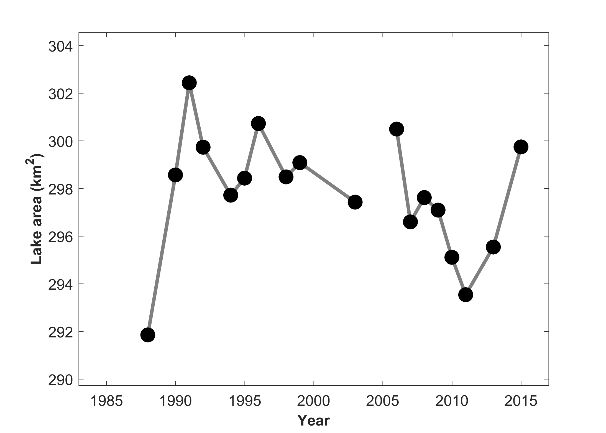


Fig. T3-5. Changes in lake area of Dianchi Lake between the mid-1980s and 2015.

Therefore, the changes in the area of Dianchi Lake were caused by both climate changes and human activities. The precipitation in the Dianchi Lake Basin has decreased during the past three decades (*12, 13*). In particular, during 2009–2012, Yunnan Province experienced a severe drought and the area of Dianchi Lake decreased obviously (Fig. T3-5). Industrial and agricultural water uses, reclamation of lakes and water diversion projects have also caused considerable influences on Dianchi Lake (*12, 13*).

*Fuxian Lake*

Fuxian Lake is another famous lake in the Yun-Gui Plateau, located in the southern part of Kunming City. The water depth of Fuxian Lake is ~87 m, and its total water volume is ~18.5 billion m^3^ (*14*). Fuxian Lake is mainly supplied by precipitation with an annual amount of 800–1,100 mm. Fuxian Lake loses water mainly through evaporation (annual evaporation is 1,200–1,900 mm) and anthropogenic consumptions (*14, 15*).


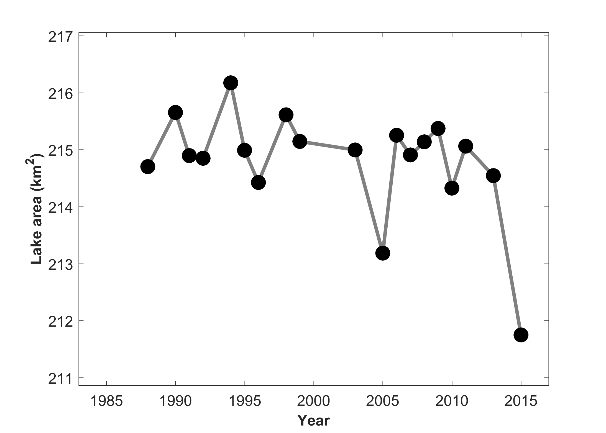


Fig. T3-6. Changes in lake area of Fuxian Lake between the mid-1980s and 2015.

The area of Fuxian Lake has been decreasing slightly in the recent decades, especially during the severe 2009–2013 drought (Fig. T3-6). According to existing researches (*14–16*), the shrinkage of Fuxian Lake was mainly caused by the reduction of rainfall and the warming temperatures. Meanwhile, human interventions, such as agricultural and construction activities, also contributed to the shrinkage of Fuxin Lake (*15*).

*Yilong Lake*

Yilong Lake, famous for its scenery, is located in the southern Yunnan Province. It is mainly supplied by precipitation and surface runoff. It loses water mainly through evaporation and human consumptions (*17*).


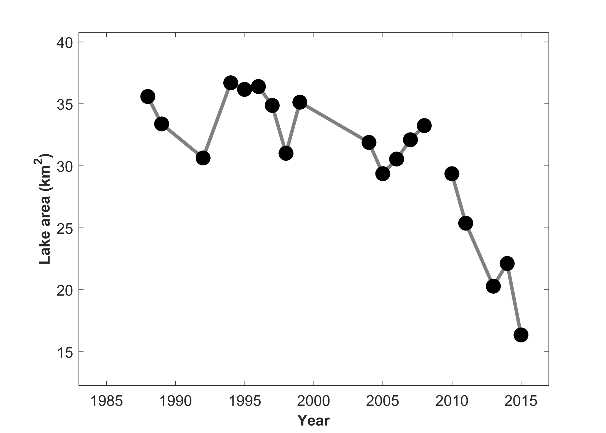


Fig. T3-7. Changes in lake area of Yilong Lake between the mid-1980s and 2015.

Due to the decreased precipitation and augmented human interventions (*18, 19*), the area of Yilong Lake has been decreasing since 1980s (Fig. T3-7). During the severe 2009–2012 drought, the area of Yilong Lake decreased obviously (Fig. T3-7). The drought also intensified the water stress of human societies around Yilong Lake. To meet the water demand of industrial and agricultural activities, Yilong Lake and its feeding rivers were heavily exploited during the drought years. Now, Yilong Lake has split into two parts, and the western part has degraded into grassland, arable land and mud flat (*18, 19*).

***Northeast Plain lake region***

Lakes in the Northeast Plain lake region are concentrated in the Songnen Plain in Northeast China. Most lakes in this region tended to shrink before 2010, which, according to existing researches, was closely related to climate changes and the construction of dams for irrigation purpose. Before 2010, the precipitation in Northeast China decreased in fluctuation while the evaporation increased (Fig. 4 in the main text). Meanwhile, the quantities of reservoirs and irrigated croplands increased largely, and there were 67 medium-sized and 413 small reservoirs within the Songnen watershed before 2005 (*20*). Since 2010, the lake area of many lakes in this region has been increasing. Most existing researches attributed the lake increase to climatic reasons such as increased precipitation.

*Chagan Lake*

Chagan Lake, located in the western Jilin Province, is one of the ten largest freshwater lakes in China (*21*). Annual precipitation at Chagan Lake (~400 mm) is much lower than annual evaporation (~1,900 mm) (*22*). The long-term average inflow is ~5.4×10^9^ m³, equal to the outflow. The inflow includes the channel lateral recharge water (1.7*10^8^ m³), the precipitation (1.45×10^8^m³), the returning flow from irrigated areas (1.31×10^8^ m³), the runoff from the Huolin River (0.96×10^8^ m³) and the recharging water from surrounding lakes or flooding areas (0.04×10^8^ m³). The outflow comprises the evaporation (3.33×10^8^ m³), the transpiration of aquatic plants (1.56 ×10^8^ m³), the leaching (0.19×10^8^ m³) and others (0.38×10^8^ m³) (*21*).


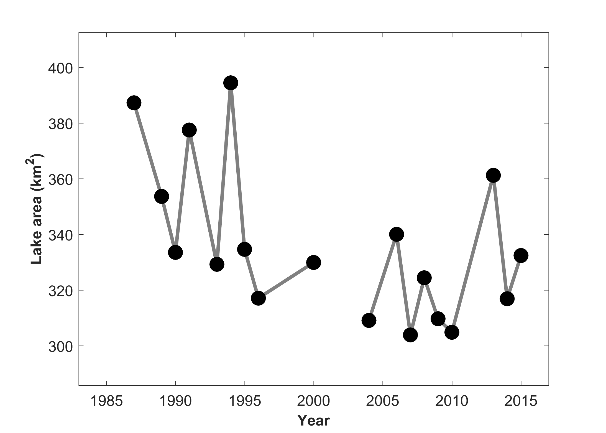


Fig. T3-8. Changes in lake area of Chagan Lake between the mid-1980s and 2015.

Before the 1960s, Chagan Lake had a vast area of ~656 km^2^ (including nearby wetlands). However, the lake shrunk sharply to 50 km^2^ at the end of the 1970s, mainly due to river interception by dams and reservoirs, as well as high intensity of evaporation. Because of the water transfer project which transferred water from the Songhuajiang River to Chagan Lake, the area of Chagan Lake increased gradually after 1976. In 1981, the area of Chagan Lake increased to 80 km^2^, and in 1986 it reached 420 km^2^ (*21*).

In the past 30 years, Chagan Lake showed an overall decreasing trend in lake area, with a slight increase after 2010 (Fig. T3-8), which was mainly caused by the climate changes (*23*). Additionally, human projects, such as ‘Returning Farmland to Forest’, also contributed to the increase of lake area by reducing agricultural water consumption (*23*).

*Xianghai Lake*

Xianghai Lake, also named as Xianghai Reservoir, is located in Jilin province. It was built in 1973 and recharged mainly by the Taoer River and the Huolin River (*24, 25*). Mean annual precipitation and annual evaporation at this lake are around 370 mm and 1,900 mm, respectively (*25*).


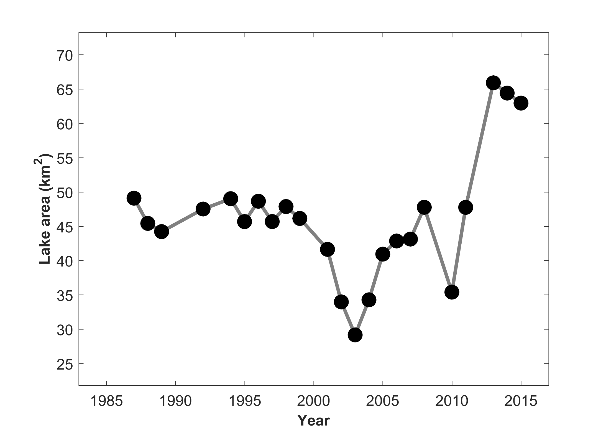


Fig. T3-9. Changes in lake area of Xianghai Lake between the mid-1980s and 2015.

Xianghai Lake decreased in area before 2003 (Fig. T3-9), because of climate changes, increased population and croplands, and human regulation of inflows of the Taoer River and the Huolin River (*24*). In the 1980s, there were about 13,000 residents in the nearby regions while it grew to over 20,000 in 2007. The area of agricultural lands increased from 6,378 ha (1988) to 8,841 ha (2007), and the populated area almost doubled compared to that of 1988 (*24*). Moreover, the inflows of Xianghai Lake’s feeding rivers were regulated by humans, which also had a profound impact on the lake (24). Recently, the area of Xianghai Lake began to increase (especially after 2010), which was believed to be caused by precipitation changes (*23*).

*Xingkai Lake*

Xingkai Lake is located in the southeastern Heilongjiang Province. It crosses the Sino-Russia boundary and ranks the largest freshwater lake in Northeast Asia (*26, 27*). Mean annual precipitation at this lake is around 600 mm and mean annual evaporation is around 490 mm (*27*). The lake is replenished mainly by rivers with a total inflow of 56×10^8^ m³. It loses water mainly through natural outflow, evaporation, leaching, and irrigation (28×10^8^ m³ in total) (*26*).


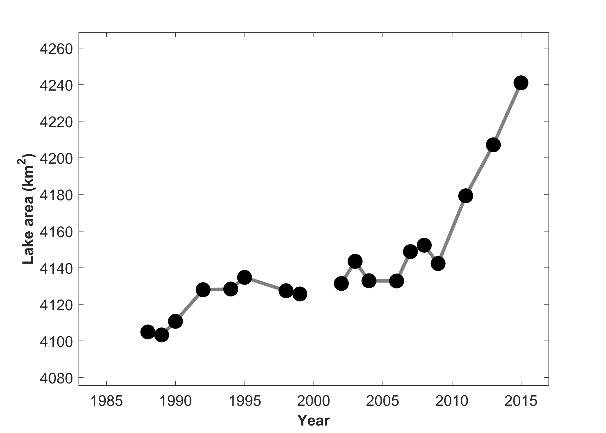


Fig. T3-10. Changes in lake area of Xingkai Lake between the mid-1980s and 2015.

The surface area of Xinkai Lake was once strongly affected by human activities in 1940s, especially by the construction of Muxing flood diversion channel (*26*). In the recent decades, Xingkai Lake was increased in area (Fig. T3-10), consistent with the increase of precipitation (*28*).

***Eastern Plain lake region***

The Eastern Plain lake region, with the middle and lower reaches of the Yangtze River (MLY) as its core region, has the largest freshwater lake cluster in China (*29*). Lakes in MLY have been influenced by humans to different extents as MLY is also one of China’s most densely populated regions. The existing researches generally agreed that the decrease in lake area in MLY was mainly caused by intensive lake impoldering, urban expansion and constructions of hydraulic engineering over the past decades.

*Taihu Lake*

Taihu Lake is one of the five largest freshwater lakes in China. The long-term averaged precipitation at Taihu Lake (21.4×10^8^–29.8×10^8^ m^3^) is slightly higher than the evaporation (20.2*10^8^–23.7*10^8^ m^3^). The water inflow into Taihu Lake (73.8×10^8^–106.4×10^8^ m^3^) is also slightly higher than the overflow (73.1×10^8^–98.6×10^8^ m^3^) (*30, 31*).


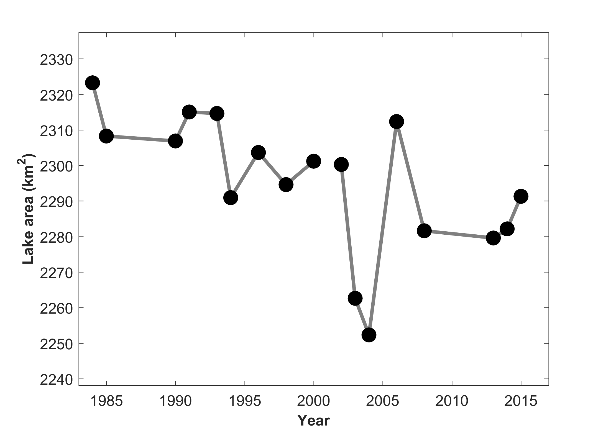


Fig. T3-11. Changes in lake area of Taihu Lake between the mid-1980s and 2015.

The area of Taihu Lake declined in the past several decades (Fig. T3-11). Shen et al (*31*) found that although the inflows, overflows, precipitation and evaporation of Taihu Lake did not change significantly, the agricultural and industrial water consumption increased from 4.5×10^8^ m^3^ (2001) to 8.5×10^8^ m^3^ (2008). Meanwhile, the Taihu Lake Basin experienced high intensity of lake impoldering during 1980–2005, and a considerable amount of lake area was changed into construction lands and grasslands (*32, 33*).

*Poyang Lake*

Poyang Lake is one of the five largest freshwater lakes of China. Mean annual precipitation and annual evaporation at Poyang Lake are ~1,570 mm and ~800 mm, respectively (*34*). Surface runoff in Poyang Lake Basin is ~1,540×10^8^ m^3^ (35) and has a dominant control on the area of Poyang Lake (*36*).


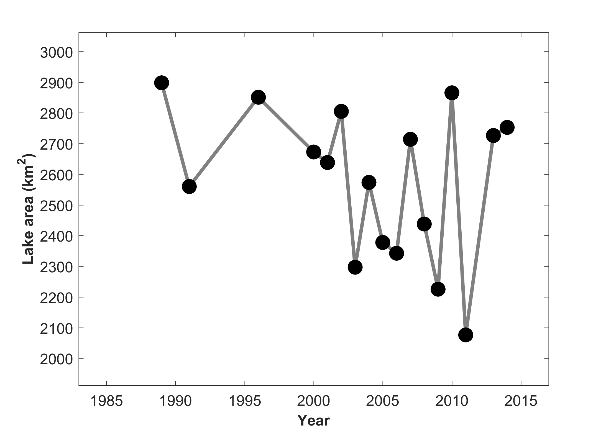


Fig. T3-12. Changes in lake area of Poyang Lake between the mid-1980s and 2015. Only Poyang Lake is shown here. Lakes connected with Poyang Lake, such as Junshan Lake, are not shown.

The area of Poyang Lake decreased with large fluctuations in the past decades (Fig. T3-12). Precipitation (*37*) and evaporation (*36, 38*) at Poyang Lake showed no significant changes during the past decades. However, there was a considerable increase in the intensity of lake impoldering (1466 km^2^ since 1949; *39*). The Three Gorges Reservoir accelerated the transition of Poyang Lake to shallow wetlands by regulating water levels in the downstream of the Yangtze River. For instance, during 1989–2006, large area of the lake degraded into grasslands (~375 km^2^), forests (~61 km^2^), beaches (~615 km^2^) and city lands (~860 km^2^) (*40*).

*Dongting Lake*

Dongting Lake is also one of the five largest freshwater lakes in China. The mean annual precipitation and annual evaporation at Dongting Lake are ~1,300 mm and ~700 mm, respectively (*41*). The inflow, overflow (as measured by Chenglingji hydrologic station) and water balance in years with a normal flow are 2925.0×10^8^ m^3^, 2605.0×10^8^ m^3^ and 320.0×10^8^ m^3^, respectively (*42*).


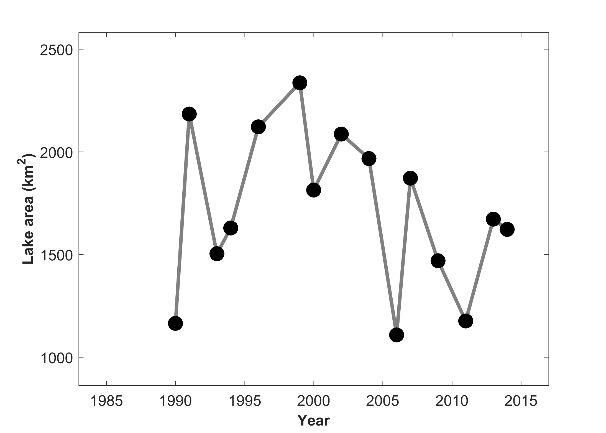


Fig. T3-13. Changes in lake area of Dongting Lake between the mid-1980s and 2015.

The area of Dongting Lake increased in the 1990s, owing to the increase in precipitation and the decrease in evaporation (*43*). After 2000, the lake area decreased significantly (Fig. T3-13), which was caused by not only the reduction of rainfall, but also human activities. Since the start of the Three Gorges Project in 2003, the total runoff as measured by Songzi, Taiping and Dianchi stations has decreased from 1457.4×10^8^ m^3^ in the 1950s to 530.6×10^8^ m^3^ in 2008, which caused a significant reduction in lake area (*44*) and the conversion of lakes into wetland vegetation (*45*). Xie et al. (*33*) also concluded that the conversion of lakes into vegetation and agricultural lands induced by human activities is a major factor influencing lake area in the Dongting Lake Basin.

***Mong-Xin Plateau lake region***

The Mong-Xin Plateau lake region is located in the arid northern part of China where lakes are heavily relied for agricultural and industrial usages. In Inner Mongolia, many lakes were suffered from river interception for irrigation and mining purposes (*46*). In Xinjiang Uygur Autonomous Region of China (Xinjiang), lake area in densely populated regions also decreased owing mainly to human activities such as irrigation (*47*).

*Hongjian Nuur*

Hongjian Nuur, or Hongjiannao Lake, is the largest freshwater lake in deserts in China. It lies on the border of Shaanxi Province and Inner Mongolia. The annual precipitation and annual evaporation at Hongjian Nuur are ~400 mm and ~1,200 mm, respectively (*48*).


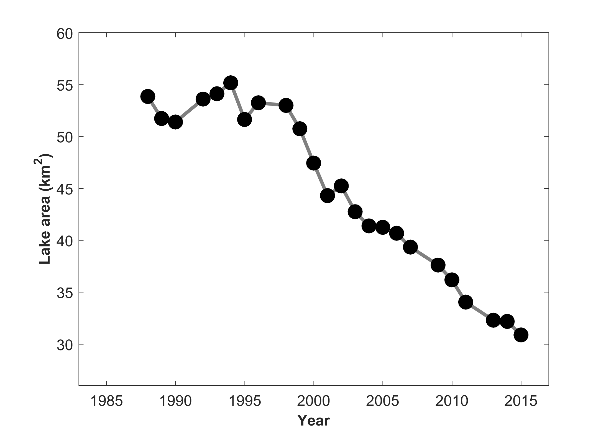


Fig. T3-14. Changes in lake area of Hongjian Nuur between the mid-1980s and 2015.

The area of Hongjian Nuur was decreased drastically since the late 1980s (Fig. T3-14). Human activities were widely believed to be the main cause of the shrinkage. Mining activities around Hongjian Nuur drained a large amount of groundwater—an important water supply for Hongjian Nuur. Meanwhile, the feeding river of Hongjian Nuur was intercepted for irrigation and mining purposes (*49*). Li et al. (*49*) concluded that climatic factors only accounted for ~ 22% of the observed lake decrease, but human activities accounted for ~78%.

*Huangqihai Lake*

Huangqihai Lake is located in the middle of Inner Mongolia, where annual precipitation is only ~ 360mm but annual evaporation is ~2,200mm, and the annual water balance of Huangqihai Lake is –0.37×10^8^ m^3^ (*29*). River inflow is thus an important factor maintaining Huangqihai Lake.


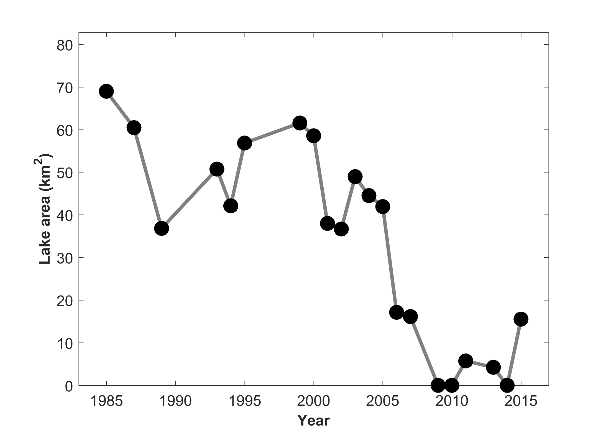


Fig. T3-15. Changes in lake area of Huangqihai Lake between the mid-1980s and 2015.

Since the late 1980s, Huangqihai Lake shrank significantly, and finally dried-up around the year of 2009 (Fig. T3-15). River interception by dams contributed largely to the dried-up of Huangqihai Lake (*29*).

*Daihai Lake*

Daihai Lake is also located in the middle of Inner Mongolia, close to Huangqihai Lake. It’s a closed inland lake with an annual precipitation of ~400 mm and annual evaporation as large as ~1,600 mm (*50, 51*).


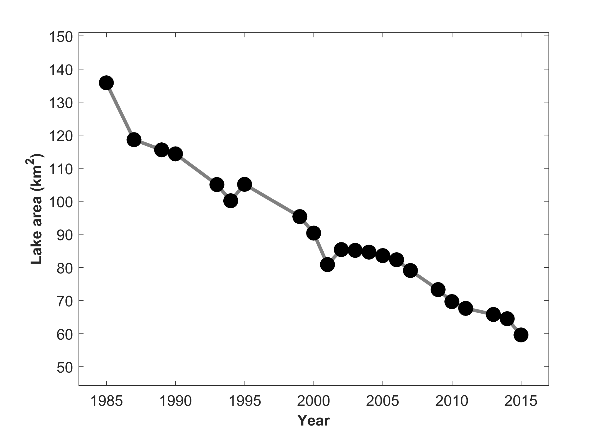


Fig. T3-16. Changes in lake area of Daihai Lake between the mid-1980s and 2015.

The area of Daihai Lake decreased by ~60% in the past decades (Fig. T3-16). Water balance analyses indicated that the decreased river inflow was the main reason of Daihai’s shrinkage (*50, 51*). Moreover, according to Liang et al. (*51*), the water consumption of agricultural and industrial activities in the Daihai Basin amounted to 63 million m^3^ in the year of 2009, but the river inflow for Daihai Lake is only 50 million m^3^. Therefore, water consumption by humans was mainly responsible for the decreased river inflow and the rapid shrinkage of Daihai Lake (*51*).

*Qehan Lake*

Qehan Lake is another typical example of human-influenced lakes in Inner Mongolia. Qehan Lake is located in the middle-northern part of Inner Mongolia, bordering with the Huanshandake Desert. The annual precipitation in this region is ~280mm, and the annual evaporation is up to ~2000mm (*52*).


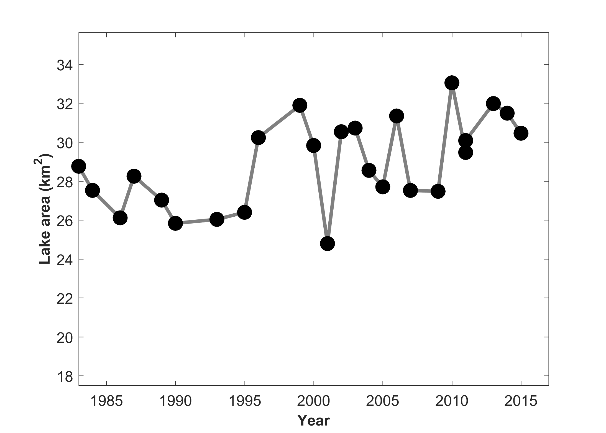

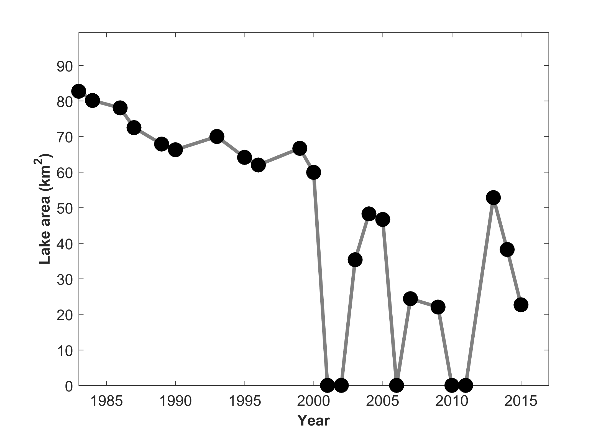


Fig. T3-17. Changes in lake area of east Qehan Lake (left) and west Qehan Lake (right) between the mid-1980s and 2015.

The area of Qehan Lake decreased in the past decades (Fig. T3-17), in part due to the drying climate, but also because of human regulation and interventions. This lake was composed of two parts: the east lake and the west lake. A dam was built between the two lakes to keep the water area of the east part for fishing purposes. As a result, the west part dried up in 2002 and turned into a source of dust storm (*52*).

*Juyan Lake*

Juyan Lake is located in the Gobi Desert in the western part of Inner Mongolia. It consists of the West Juyan lake and the East Juyan Lake, both receiving the water of the Heihe River as the main water supply (*53*).


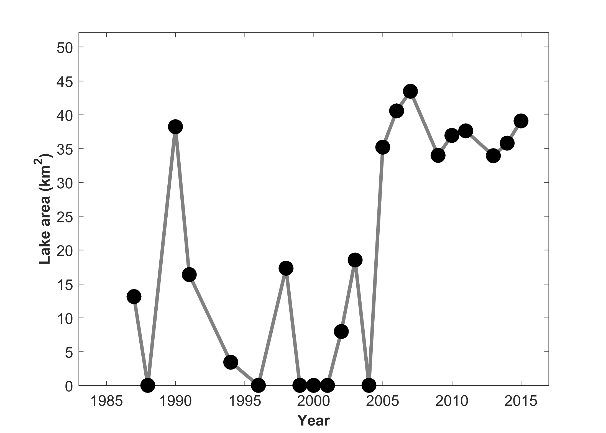


Fig. T3-18. Changes in lake area of east Juyan Lake between the mid-1980s and 2015.

Due to high intensity of human activities in the middle reaches of the Heihe River, such as river interception for irrigation, the West Juyan Lake dried up in 1962 (*53, 54*). The East Juyan Lake also dried up completely in 2000 (Fig. T3-18). Because of the water diversion project launched by the local government (*53, 54*), the area of the East Juyan Lake returned to ~37 km^2^ recently (Fig. T3-18).

*Wulagai lake Group*

The Wulagai Lake Group is located in the Wulagai Grassland in the northern part of Inner Mongolia. It contains several lakes with area greater than 10 km^2^ and many small lakes. These lakes are mainly fed by the Wulagai River. However, the Wulagai River was intercepted by a reservoir for providing water for the nearby coal mines (*55, 56*). The Wulagai Lake Group therefore completely dried up in 2004 (Fig. T3-19). After 2010, with the increase of local precipitation, the area of the Wulagai Lake Group increased to a small extent (Fig. T3-19).


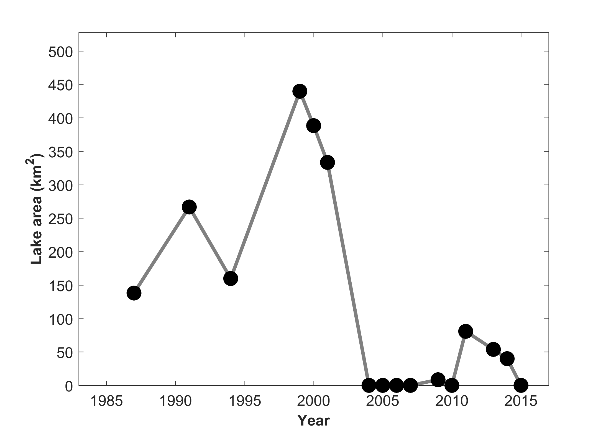

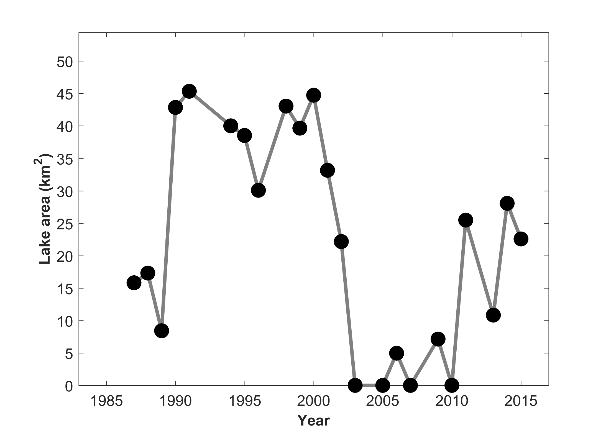


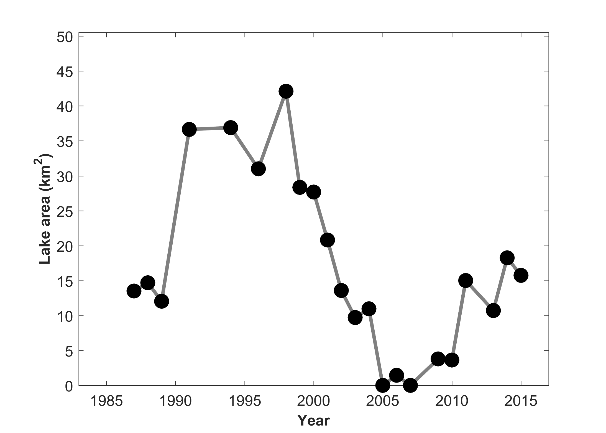

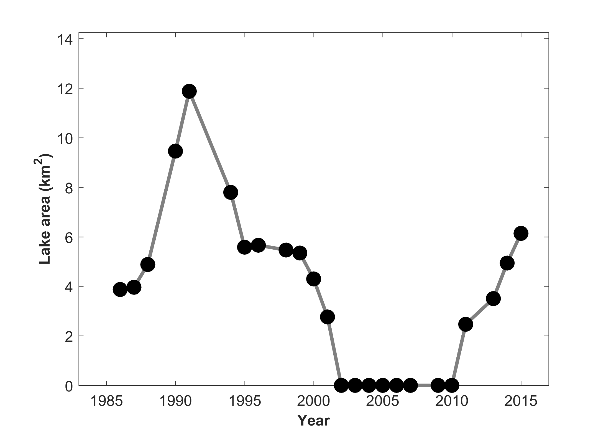


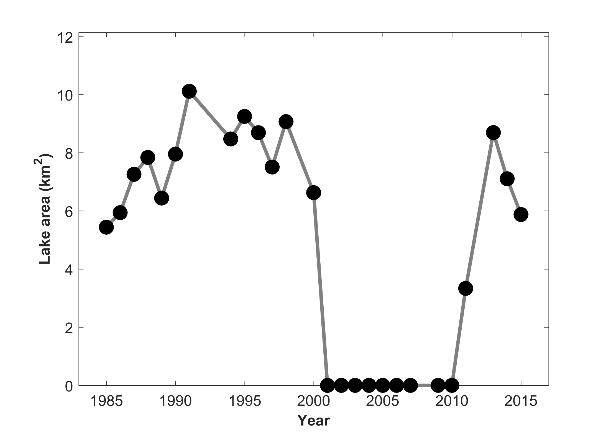

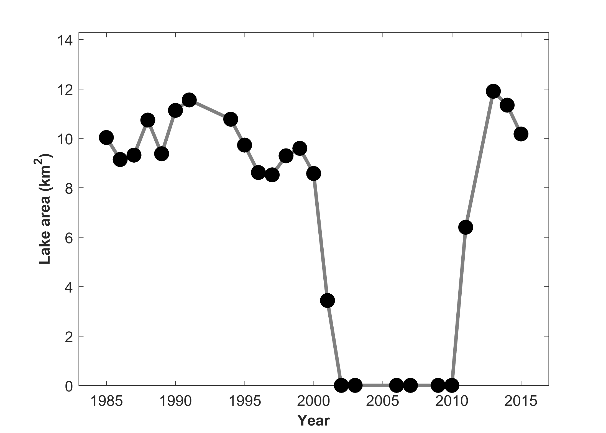


Fig. T3-19. Changes in lake area of six major lakes in the Wulagai Lake Group between the mid-1980s and 2015.

*Sayram Lake*

Sayram Lake is the largest mountain lake in the northwestern part of Xinjiang. This lake is less impacted by human interventions due to its unique location in the Mt. Tianshan. Sayram Lake is mainly fed by mountain rivers, springs and a number of seasonal rivers. The total runoff from rivers and springs is ~0.94×10^8^ m^3^. Sayram Lake loses water mainly through evaporation, and through percolation from the bottom of the lake to the groundwater (*57*).


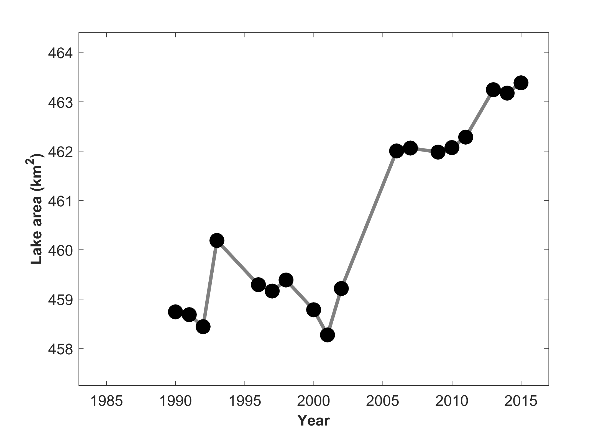


Fig. T3-20. Changes in lake area of Sayram Lake between the mid-1980s and 2015.

Sayram Lake gained its area in the past decades (Fig. T3-20). Temperatures in the Sayram Lake Basin gradually increased since the 1980s, which not only enhanced lake evaporation but also provided glacial meltwater for the lake. Meanwhile, precipitation in this region also increased. The changes in meltwater and precipitation increased river runoff and finally led to the expansion of Sayram Lake (*58, 59*).

*Ulungur Lake and Jili Lake*

Ulungur Lake and Jili Lake are located in north Xinjiang. Ulungur Lake is the terminal lake of the Ulungur River and connects to the Jili lake through Kuyiga River. A dam was built on the Kuyiga River in 1972 to control the water level of Jili Lake. The mean annual precipitation around Ulungur Lake is ~120 mm and the annual evaporation is ~1,200 mm (*60*). The total amount of inflow is ~8.0×10^8^ m^3^, among which ~2.8×10^8^ m^3^ was provided by the Ulungu River. The mean annual evaporation around Ulungur Lake and Jili Lake is ~6.5×10^8^ m^3^ and ~1.5×10^8^ m^3^, respectively (*61*).


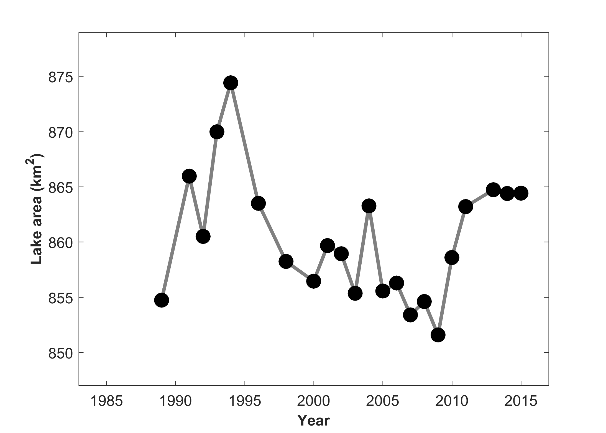

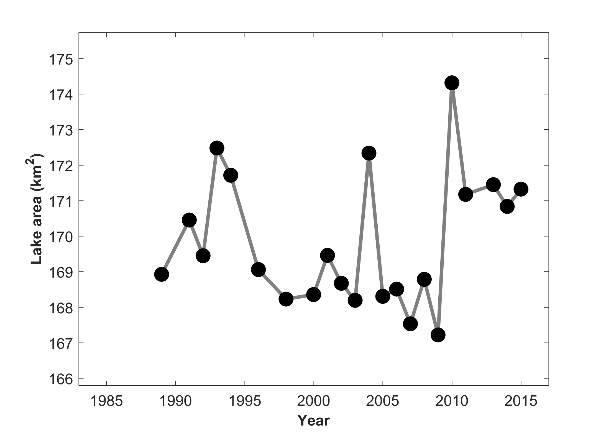


Fig. T3-21. Changes in lake area of Ulungur Lake (left) and Jili Lake (right) between the mid-1980s and 2015.

The area of the two lakes increased during the late 1980s–1990s (Fig. T3-21), consistent with the changes in local precipitation. Starting from the year of 2000, the runoff of the Ulungur River decreased due to climate changes and agricultural irrigation (*62*), which resulted in a dramatic decrease in the lake area (Fig. T3-21). Therefore, climate changes and human activities both had considerable controls on the area of the two lakes (*62*).

*Bosten Lake*

Bosten Lake is an inland freshwater lake in the southern foothills of Mt. Tianshan in central Xinjiang. The annual inflow, outflow, precipitation and evaporation of this lake are 16.0×10^8^ m^3^, 8.1×10^8^ m^3^, 0.8×10^8^ m^3^, 8.2×10^8^ m^3^, respectively. The Kaidu River provides the largest perennial inflow into Bosten Lake (*63*).


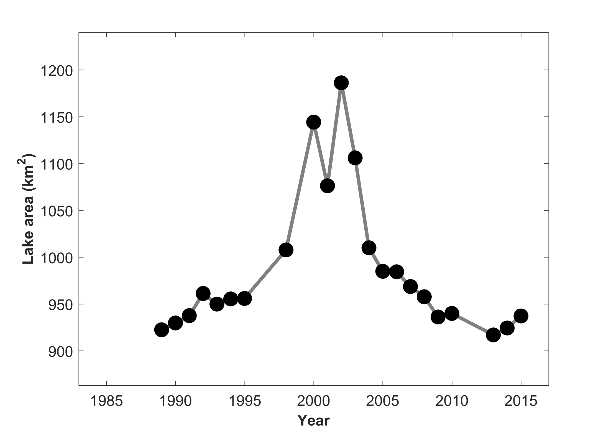


Fig. T3-22. Changes in lake area of Bosten Lake between the mid-1980s and 2015.

Between 1988–2002 and 2002–2010, the rapid expansion and shrinkage of Bosten Lake (Fig. T3-22) were mainly caused by variations in the runoff of the Kaidu River (*63*). Both climate changes and human activities impacted the runoff (*63*). Climate changes controlled the runoff of Kaidu River by providing meltwater from glaciers and snow (*64*). Human irrigation was estimated to consume 27.1%–36.5% of total runoff of the Kaidu River (*65*). Quantitative analysis showed that climate changes and human activities explained 30.4% and 69.6% of the variations in runoff, respectively (*66*).

References

1. Li, X., Xu, H., Sun, Y., Zhang, D., Yang, Z. Lake-Level change and water balance analysis at Lake Qinghai, West China during recent decades.*Water Resour Manag* **21**,1505–1516 (2007).

2. Shi, X., Li, Y., Li, S., Qin, N., Su, Z. Calculation on the variables of water amount of Qinghai Lake, and their variations. *Qinghai Meteorol* **S1**,72–79 (2008). [In Chinese]

3. Zhang, G., Xie, H., Yao, T., Kang, S. Water balance estimates of ten greatest lakes in China using ICESat and Landsat data. *Chini Sci Bull* **58**, 3815–3829 (2013).

4. Zhang, G., Xie, H., Yao, T., Li, H., Duan, S. Quantitative water resources assessment of Qinghai Lake basin using Snowmelt Runoff Model (SRM). *J Hydrol* **519**, 976–987 (2014).

5. Pi, Y., Liu, S., Li, Z., Xin, R., Zhang, X. Remote-sensing survey and dynamic change analysis on lakes in Qinghai Province based on GF-1 and Landsat satellite data. *J Ningxia Univ* **39**,170–176 (2018). [In Chinese]

6. DeJi, Y., Nima, J., Qiangba, O., Zeng, L., Luosang, Q. Lake area variation of Selin Tso in 1975–2016 and its influential factors. *Plateau Mt Meteorol Res* **38**, 35–41 (2018). [In Chinese]

7. Bian, D., Bian, B., La, B., Wang, C., Chen, T. The response of water level of Selin Co to climate change during 1975–2008. *Acta Geol Sin* **65**, 313–319 (2010). [In Chinese]

8. Meng, K., Shi, X., Wang, E., Liu, F. High-altitude salt lake elevation changes and glacial ablation in Central Tibet, 2000–2010. *Chini Sci Bull* **57**, 525–534 (2012).

9. Wu, Y., Zhu, L., Ye, Q., Wang, L. The response of lake-glacier area change to climate variations in Namco Basin, Central Tibetan Plateau, during the last three decades. *Acta Geol Sin* **62**, 301–311 (2007). [In Chinese]

10. Ma, Y., Yi, C., Wu, J., Jin, Y. Lake surface expansion of Nam Co during 1970–2009: Evidence of satellite remote sensing and cause analysis. *J Glaciol Geocryol* **34**, 81–88 (2012). [In Chinese]

11. Zhi, G. *et al*. Comprehensive balance management of water resources in the Dianchi Lake Basin. *Res* *Environ Yangtze Basin* **22**, 1227–1233 (2013). [In Chinese]

12. Xiao, Q., Yang, K., Hong, L. Remote sensing monitoring and temporal-spatial analysis of surface water body area changes of lakes on the Yunnan-Guizhou Plateau over the past 30 years. *J Lake Sci* **30**,1083–1096 (2018). [In Chinese]

13. Li, H., Chong, D., Fan, S., Zhang, S., Wang, J. Remote sensing monitoring of the nine plateau lakes' surface area in Yunnan in recent thirty years. *Res* *Environ Yangtze Basin* **25**, 32–37 (2016). [In Chinese]

14. Shi, Z., Wen, X., Ma, W., Shen, P. Application of tasseled cap transformation in Fuxian Lake area changes analysis. *J Anhui Agr Sci* **43**, 352–355 (2015). [In Chinese]

15. Du, S., Jin, B., Li, S., Zhou, J., Peng, S. The spatial-temporal variation of Fuxian Lake area during 1974–2014 based on remote sensing. *Oceanol Limnol* *Sin* **49**, 40–51 (2018). [In Chinese]

16. Wang, X., Li, S. Driving force analysis of the area and volume change of Fuxian Lake in the last 40 years. *J Yuxi Normal Univ* **33**, 36–42 (2017). [In Chinese]

17. Li, S. Ecological environmental integrated control for Yilonghu watershed. *For Inven Plan* **34**, 108–110 (2009). [In Chinese]

18. Xiao, Q., Yang, K., Cao, L., Hong, L. Water body extraction and lake area dynamic monitoring of Qilu Lake and Yilong Lake. *Anhui Agr Sci Bull* **23**, 123–124 (2017). [In Chinese]

19. Xiao, Q., K. Yang, and L. Hong. 2018. Remote sensing monitoring and temporal-spatial analysis of surface water body area changes of lakes on the Yunnan-Guizhou Plateau over the past 30 years. *J Lake Sci* **30**, 1083–1096. [In Chinese]

20. Jiang, M., Lv X. G., Xu, S. L., Tong, S. Z. Perturbation factors and feedback of wetland ecosystem in the Songnen Plain. *Resour Sci* **6**, 125–131 (2005) [In Chinese]

21. Li, X. S. Study on the health assessment of Chagan Lake. Master thesis, Jilin University, Shenyang, 2018 [In Chinese]

22. Sun K. J. Study on the balance between water supply and recharge in Chagan lake. *Jilin Water Conserv* **6**, 1–4 (2014) [In Chinese]

23. Ma, Y. M. *et al.* Remote sensing monitoring on area dynamic change of major water bodies in western Jilin Province. *Bull Soil Water Conserv* **5**, 249–255 (2018) [In Chinese]

24. Lang, Z. H., Xu, M. X., Gao, H. W. Water environment situation and protection measures for Xianghai wetland in Jilin Province. *Yantze River* **5**, 47–49 (2011) [In Chinese]

25. Wang Y. L. A Study on ecological compensation mechanism of Xianghai wetland water supplement. PhD thesis, Jilin University, Shenyang, 2012. [In Chinese]

26. Ji, Z. G., Wu, M. G. Preliminary study on the availability of water resources in the Sino-Russia boundary lake, Xingkai Lake. *Heilongjiang Sci Technol Water Conserv* **2**, 47–49 (2006) [In Chinese]

27. Zhu, Y., Shen, J., Lei, G., Wang, Y. Environmental evolution of Xingkai (Khanka) Lake since 200 ka by OSL dating of sand hills. *Chin Sci Bull* ***56***, 2604–2612 (2011).

28. Li, N., Liu, J. P., Wang, Z. M. Dynamics and driving force of lake changes in northeast China during 2000–2010. *J Lake Sci* **4**, 545–551 (2014) [In Chinese]

29. Wang, S. M., Dou. H. S. Lakes in China (Science Press, Beijing, 1998). [In Chinese]

30. Ge, L. B. Water inflow and outflow and water balance in Taihu Lake. *Jiangsu Water Resour* **11**, 33–34 (2007). [In Chinese]

31. Shen, J*.* Y*. et al.* Taihu Lake Water Balance Factors and Error Control Measures. *J China Hydrol* **31**, 60–63 (2011). [In Chinese]

32. Zhang, X. Z. *et al.* Analysis of land use and landscape pattern change in the Taihu Lake region in China in the past 20 years. *China Land Association Academic Annual Meeting* (2008). [In Chinese]

33. Xie, C. *et al.* Impacts of Land-Use Changes on the Lakes across the Yangtze Floodplain in China. *Environ Sci Technol* **51**, 3669–3677 (2017).

34. Zhao, X. S., Liu, Y. B., Wu, G. P. A remote-sensing-based study on evapotranspiration and the environmental factors over the Lake Poyang region. *J Lake Sci* **25**, 428–436 (2013). [In Chinese]

35. Zhu, L. M. *et al*. Simulation and analysis of water balance process in Poyang Lake Basin based on system dynamic approach. *Water Resour Prot* **31**, 46–52 (2015). [In Chinese]

36. Luo, W., Zhang, X., Deng, Z. M., Xiao, Y. Variation of the total runoff into Poyang lake and drought-flood abrupt alternation during the past 50 Years. *J Basic Sci Eng* **21**, 845–856 (2013). [In Chinese]

37. Yin, J. M. *et al.* Analysis of climate change law in Poyang Lake Basin in the past 50 years. Chinese Meteorological Society. *Regional Climate Change Monitoring and Detection Symposium* (2011). [In Chinese]

38. Min, Q., Su, Z. P., Wang, S. J. Characteristics and causes of Poyang Lake surface evaporation variation in recent 50 years. *Meteor Disast Re Res* **30**, 17–20 (2007). [In Chinese]

39. Dou, H. S., Min, Q., Shi, F. X. Impacts of reclamation on the flood regime in Poyang Lake and countermeasures. *J Lake Sci* **11**, 20–27 (1999). [In Chinese]

40. Zhou, Y. K., Bai, X. L., Jiang, J. H. Dynamic land use changes in the Poyang Lake region over the recent 17 Years. *Resour Sci* **33**,1186–1194 (2011). [In Chinese]

41. Peng, J., Li, G., Wu, F. Analysis on the Changes of Utilizable Precipitation over Dongting Lake Region during the Past 100 Years. *Ecol Environ Sci* **26**: 104–110 (2017).

42. Zhou, L. *et al.* Dynamics of water level of Lake Dongting during the past 60 years and the associated driving factors. *J Glaciol Geocryol* **39**, 210–221 (2017). [In Chinese]

43. Wang, G. *et al*. Characteristic of climate change in the Lake Dongting Basin (1961–2003). *J Lake Sci* **18**, 470–475 (2006). [In Chinese]

44. Yuan, M. *et al*. Effects of the Three Gorges Project on the water surface area of Lake Dongting, 2000–2010. *J Lake Sci* **26**, 37–45 (2014). [In Chinese]

45. Cui, L. *et al.* Dynamics of the lakes in the middle and lower reaches of the Yangtze River basin, China, since late nineteenth century. *Environ Monit Assess* **185**, 4005–4018 (2013).

46. Tao, S. L. *et al.* Rapid loss of lakes on the Mongolian Plateau. *Proc Natl Acad Sci USA* **112**, 2281–2286 (2015).

47. Fang, L. Q., Tao, S. L., Zhu, J. L., Liu, Y. Impacts of climate change and irrigation on lakes in arid northwest China. *J Arid Environ* **154**, 34–39 (2018).

48. Tang, K., Wang, H., Liu, C. Preliminary study of Hongjiannor Lake’s variation and ecologicla water demand. *J Nat Resour* **18**, 304–309 (2003).

49. Li, D., Zhuo, J., Wang, Z. Effect of human activities and climate change on the water surface area of Hongjiannao Lake*.* *J Glaciol Geocryol* **31**: 1110–1115 (2009). [In Chinese]

50. Zhou, Y., Jiang, J. Changes in ecological environment in the Daihai Lake Basin over the last 50 years. *Arid Zone Res* **26**, 162–167 (2009). [In Chinese]

51. Liang, W. *et al*. Research on the area change processes in the past 40a of Daihai Lake. *J Arid Land Resour Environ* **31**, 93–98. 2017. [In Chinese]

52. Liu, M., Hasi, E., Chun, X. Variation and causation of Lake Qehan, Inner Mongolia over the recent 50 years. *J Lake Sci.* **27**, 141–149 (2015). [In Chinese]

53. Nian, Y., Li, X., Zhou, J., Hu, X. Impact of land use change on water resource allocation in the middle reaches of the Heihe River Basin in northwestern China. *J Arid Land* **6**, 273–286 (2014).

54. Cheng, G. *et al.* Integrated study of the water–ecosystem–economy in the Heihe River Basin. *Natl Sci Rev* **1**, 413–428 (2014).

55. Zhang, B., Song, X., Ma, Y., Bu, H. Impact of coal power base constructions on the environment around the Wulagai water reservoir, Xilinguole, Inner Mongolia. *J Arid Land Resour Environ* **27**,190–194 (2013). [In Chinese]

56. Song XF, Bu HM, Ma Y, eds (2012) [*Thirsty coal:coal power base constructions and water resource*] (China Environmental Science Press,Beijing)

57. Wang, S. J. River and Lake in Xinjiang, China. Beijing: China. *Water Resources and Hydropower Press* (2011). [In Chinese]

58. Wang, Y., *et al.* Time-series analysis of Sayram Lake area changes during 1989–2014. *Arid Land Geogr* **39**, 851–860 (2016). [In Chinese]

59. Ma, D. *et al.* Influence of the warm-wet climate on Sayram Lake. *J Glaciol Geocryol* **25**, 219–223 (2003). [In Chinese]

60. Wu, J. L., Ma, L., Ceng, H. A. Water quantity and quality change of Ulungur Lake and its environmental effects. *J Na Res* **28**, 844–853(2013). [In Chinese]

61. Liu, J. J. Analysis on the water amount flowing into Ulungur Lake. *Energy Energy Conserv* (5), 103–105 (2015). [In Chinese]

62. Huang, Z. H., *et al.* The impact of human activities on the environmental evolution of Ulungur Lake. *Yellow River* **33**, 60–62 (2011). [In Chinese]

63. Ilnur, G. *et al.* The driving forces of the Bosten Lake water level variations in 1958–2012. *J Desert Res* **35**, 240–247 (2015). [In Chinese]

64. Ma, L. J. *et al.* Impact of glacier and snow melting on Bosten Lake under climate change. *Arid Land Geogr* **33**, 201–216 (2010). [In Chinese]

65. Gao, Z. H., Zhu, C., Li, Z. R. Quantitative analysis of the impact of irrigation water from Kaidu River on the area change of Bosten Lake. *J Nat Res* **20**, 502–507 (2005). [In Chinese]

66. Wang, J., Chen, Y. N., Chen, Z. S. Quantitative assessment of climate change and human activities impact the inflowing runoff of Bosten Lake. *Xinjiang Agr Sci* **49**, 581–587 (2012). [In Chinese]

**Supplementary Text 4**

*Case studies on the protections of China’s lakes*

Lake restoration actions are being implemented across China. Here we presented four examples, including Juyan Lake, Hongjian Nuur, Napa Lake, and Baiyangdian Lake (see Fig. T4-1 for their locations).


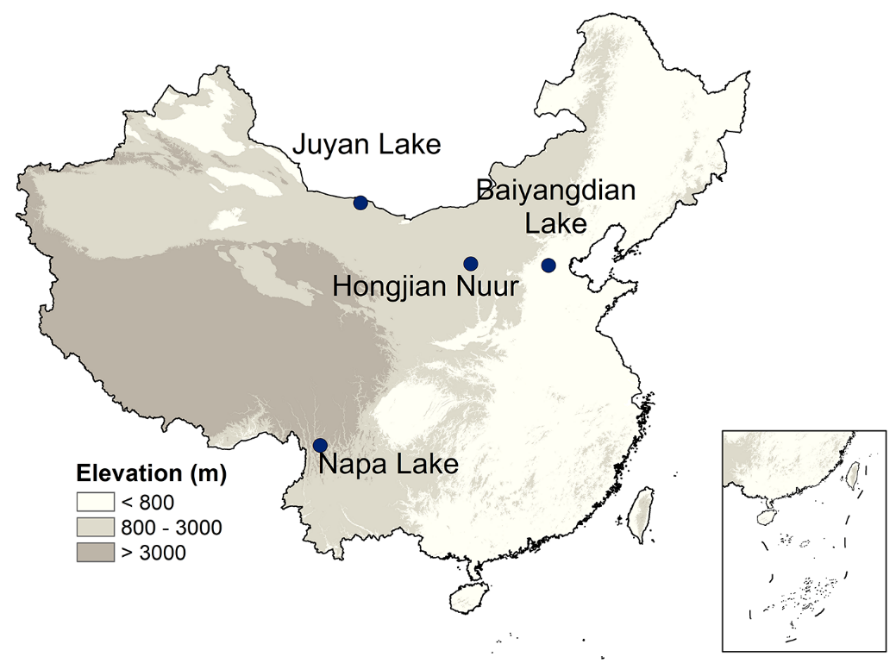


Fig. T4-1. Locations of four typical lakes which are being restored by human projects.

*Juyan lakes*

Juyan lakes, located in the Gobi Desert in the western part of Inner Mongolia, are the terminal lakes of the Zhangye Heihe Wetland—one of the Ramsar international important wetlands (*1*). Juyan lakes comprise the West Juyan lake (Gashuun Nuur in Mongolian) and the East Juyan Lake (Sogo Nuur in Mongolian). The past decades witnessed a drastic increase in population and human activities around the two lakes. It was estimated that human irrigation interpreted 75% of the annual runoff of the Heihe river (13.0×10^8^ out of 17.3×10^8^ m^3^/a) (*2*), causing directly the dried-up of the Juyan lakes. Since the year of 2000, the local government launched a water diversion project aiming at restoring the two lakes. After several times of water diversion conducted between 2002 and 2006 (*3*), the area of the Juyan lake returned to ~37 km^2^ recently.

*Hongjian Nuur*

Hongjian Nuur (Nuur means lake in Mongolian) is in the Maowusu Desert in the Mong-Xin lake region. Hongjian Nuur is managed by two provinces, Shaanxi Province and Inner Mongolia. It is the largest desert freshwater lake in China with a stunning scenery. Moreover, it is one of the largest habitats of relict gulls, a national first-class endangered bird species. However, the most important incoming river of Hongjian Nuur was intercepted to meet the water demand of farmland irrigation and coal-related industries (*4*). Our calculation indicated that the lake area of Hongjian Nuur changed from 54 km^2^ (1988) to 31 km^2^ (2015). Fortunately, according to some media reports, the governments of Shaanxi Province and Inner Mongolia launched several projects to save Hongjian Nurr.

*Napa Lake*

Napa Lake (or Napa wetland), located in Shangri-la in the Yun-Gui Plateau, is a Ramsar international important wetland with a stunning natural scenery (*1*). It’s an important habitat of Black-necked crane (*Grus nigricollis*), a species with a global population of around 8000 individuals and categorized as vulnerable according to the IUCN Red List (*5*). However, the lake was decreased in size under the pressures from population growth, grazing and tourism (*6*). Starting from 2015, the local government has reinforced the management of impoldering and launched a water diversion project to restore Napa lake.

*Baiyangdian lake*

This lake is in Hebei Province, known as the largest freshwater lake in the North China Plain. Since the 1950s, it was suffered from huge pressures from drying climate and human interventions such as over-exploitation of groundwater and water pollution (*7*). The protection and restoration of Baiyandian Lake was one of the priorities of the local governments, and water diversion projects started as early as in 1974. In 2017, Chinese Central Government established Xiong’an New Area, a state-level new area located on the southwest of Beijing. Baiyangdian Lake lies at the center of the Xiong’an New Area and is now being restored with great efforts.

References

1. Ramsar Convention Bureau, The List of Wetlands of International Importance (The Secretariat of the Convention on Wetlands, Gland, Switzerland, 2017).
2. Nian, Y., Li, X., Zhou, J., Hu, X. Impact of land use change on water resource allocation in the middle reaches of the Heihe River Basin in northwestern China. *J Arid Land* **6**, 273–286 (2014).
3. Cheng, G.D. *et al.* Integrated study of the water–ecosystem–economy in the Heihe River Basin. *Natl Sci Rev* **1**, 413–428 (2014).
4. Li, D., Zhuo, J., Wang, Z. Effect of human activities and climate change on the water surface area of Hongjiannao Lake. *J Glaciol Geocryol* **31**, 1110–1115 (2009). [In Chinese]
5. Liu, Q., Yang, J., Yang, X., Zhao, J., Yu, H. Foraging habitats and utilization distributions of Black-necked Cranes wintering at the Napahai Wetland, China. *J Field Ornithol.* **81**, 21–30 (2010). [In Chinese]
6. Wu, G., Gao, Y., Wang, Y., Wang, Y., Xu, D. Land-use/land cover changes and their driving forces around wetlands in Shangri-La County, Yunnan Province, China. *Int J Sust Dev World* **22**, 110–116 (2015).
7. Hu, S., Liu, C., Zheng, H., Wang, Z., Yu, J. Assessing the impacts of climate variability and human activities on streamflow in the water source area of Baiyangdian Lake. *J Geogr Sci* **22**, 895–905 (2012).
